# Precise Design of TiO_2_@CoO_x_ Heterostructure via Atomic Layer Deposition for Synergistic Sono‐Chemodynamic Oncotherapy

**DOI:** 10.1002/advs.202304046

**Published:** 2024-02-04

**Authors:** Wen Liu, Runrun Shao, Lingyun Guo, Jianliang Man, Chengwu Zhang, Lihong Li, Haojiang Wang, Bin Wang, Lixia Guo, Sufang Ma, Bin Zhang, Haipeng Diao, Yong Qin, Lili Yan

**Affiliations:** ^1^ Basic Medical College Shanxi Medical University Taiyuan 030001 P. R. China; ^2^ Key Laboratory of Cellular Physiology at Shanxi Medical University Ministry of Education Taiyuan 030001 P. R. China; ^3^ Pharmacy College Shanxi Medical University Taiyuan 030001 P. R. China; ^4^ State Key Laboratory of Coal Conversion Institute of Coal Chemistry Chinese Academy of Sciences Taiyuan 030001 P. R. China

**Keywords:** atomic layer deposition, carrier separation efficiency improvement, energy band structure adaptation, heterostructure, synergistic sono‐chemodynamic oncotherapy

## Abstract

Sonodynamic therapy (SDT), a tumor treatment modality with high tissue penetration and low side effects, is able to selectively kill tumor cells by producing cytotoxic reactive oxygen species (ROS) with ultrasound‐triggered sonosensitizers. N‐type inorganic semiconductor TiO_2_ has low ROS quantum yields under ultrasound irradiation and inadequate anti‐tumor activity. Herein, by using atomic layer deposition (ALD) to create a heterojunction between porous TiO_2_ and CoO_x_, the sonodynamic therapy efficiency of TiO_2_ can be improved. Compared to conventional techniques, the high controllability of ALD allows for the delicate loading of CoO_x_ nanoparticles into TiO_2_ pores, resulting in the precise tuning of the interfaces and energy band structures and ultimately optimal SDT properties. In addition, CoO_x_ exhibits a cascade of H_2_O_2_→O_2_→·O_2_^−^ in response to the tumor microenvironment, which not only mitigates hypoxia during the SDT process, but also contributes to the effect of chemodynamic therapy (CDT). Correspondingly, the synergistic CDT/SDT treatment is successful in inhibiting tumor growth. Thus, ALD provides new avenues for catalytic tumor therapy and other pharmaceutical applications.

## Graphical abstract

Heterostructures precisely designed and constructed by atomic layer deposition technology have been used in in vivo experiments for the first time. The interfacial and energy band structure of TiO_2_@CoO_x_ heterojunction can be finely tuned by gradient ALD of CoO_x_ on TiO_2_, resulting in a synergistic effect between sonodynamic and chemodynamic therapy.

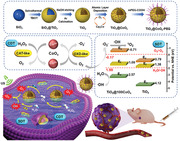

## 1 Introduction

Sonodynamic therapy (SDT), a stimulation response treatment method, can specifically injure cancer cells by inducing the production of cytotoxic reactive oxygen species (ROS) via sonosensitizers that are activated by ultrasound.^[^ ^1^ ^]^ With greater tissue penetration (7–10 cm) and fewer side effects than near‐infrared (NIR) light, SDT has incomparable advantages over other stimulated response treatment methods for deep tumor treatment.^[^ ^2^ ^]^ It has been reported that SDT has the potential to cure patients with advanced breast cancer (invasive ductal carcinoma, grade 3),^[^ ^3^ ^]^ advanced non‐small cell lung cancer (lung adenocarcinoma, stage 3B),^[^ ^4^ ^]^ and advanced intrahepatic cholangiocarcinoma,^[^ ^5^ ^]^ without significant side effects. However, most sonosensitizers used in clinical oncology are organic porphyrins and their derivatives, which always suffer from skin sensitivity, inadequate stability, and unfavorable tumor accumulation, thereby limiting SDT development in clinical oncology.^[^ ^6^ ^]^

Inorganic TiO_2_ nanosonosensitizer is a novel sonosensitizer with ultrasound (US) stability, biocompatibility, and tumor specificity superior to organic sonosensitizers molecules.^[^ ^7^ ^]^ Under US stimulation, electrons on the valence band (VB) of TiO_2_ may move to the conduction band (CB), resulting in the formation of electrons and holes that react with the surrounding O_2_ and H_2_O molecules to produce cytotoxic ROS (such as ^1^O_2_, ·O_2_^−^, and ·OH) and induce cell apoptosis or necrosis.^[^ ^8^ ^]^ Consequently, the therapeutic effect of these TiO_2_ is highly dependent on the US‐generated electron–hole pairs separation efficiency, also known as the carriers separation efficiency. Porous TiO_2_ exhibited superior sonocatalytic properties than non‐porous TiO_2_ due to its higher active site concentration. However, pure TiO_2_ remains deficient in US‐triggered ROS production efficiency for its wide band gap, mismatched energy band structure, and rapid electron‐hole pairs recombination, resulting in insufficient anti‐tumor activity.^[^ ^9^ ^]^ Hence, efforts have been made to improve carrier separation and SDT efficiency of TiO_2_ by creating oxygen vacancies,^[^ ^10^ ^]^ doping and atoms such as Fe and W in their lattice,^[^ ^11^ ^]^ and decorating metal or metal compounds (such as Au,^[^ ^12^ ^]^ Pt,^[^ ^13^ ^]^ Fe_3_O_4_,^[^ ^14^ ^]^ CuS,^[^ ^15^ ^]^ etc.) on the surface to form heterojunctions. However, since O_2_ is a reactant in the CB reaction and the tumor microenvironment (TME) is hypoxic, the sonosensitizers prepared by these strategies are still inefficient for ROS generation under US irradiation.

Cobalt oxide (CoO_x_) decoration TiO_2_ is a promising alternative. It has been demonstrated that by bridging CoO_x_ onto porous TiO_2_, heterojunctions can be formed and the electron distribution in the system can be modified, thereby significantly inhibiting the recombination of electron‐hole pairs and enhancing the sonocatalytic oxidation activity.^[^ ^16^ ^]^ In addition, CoO_x_ exhibited a cascade of catalase (CAT)‐like and oxidase (OXD)‐like activities, which exhibited a cascade of H_2_O_2_→O_2_→·O_2_^−^ in response to TME.^[^ ^17^ ^]^ Thus, decorating CoO_x_ on porous TiO_2_ not only effectively constructs heterojunctions and improves SDT performance, but also ameliorates the hypoxia during the SDT process. In addition, CoO_x_ can induce the chemodynamic therapy (CDT) effect. Consequently, the design and synthesis of highly efficient TiO_2_@CoO_x_ nanoparticles and their application in synergistic SDT/CDT are anticipated to lead to new advances in treating malignant tumors. Unfortunately, few studies have been reported on TiO_2_–CoO_x_ heterojunction construction and their CDT/SDT effects on tumors to date.

Although coating with CoO_x_ may improve the SDT performance of porous TiO_2_, the extent of this improvement is dependent on the interface interaction between CoO_x_ and TiO_2_, particularly particle loading amount and complex interface state.^[^ ^18^ ^]^ Traditional loading methods, such as impregnation and chemical precipitation, have weak interactions, poor controllability, and insufficient step coverage for precisely loading CoO_x_ into TiO_2_ pores, making SDT performance optimization a formidable challenge. Atomic layer deposition (ALD) is a nanomaterial preparation technique with atomic‐level precision.^[^ ^19^ ^]^ Given the unparalleled advantages of other methods in fine structure preparation, the ALD technique enables precise loading of CoO_x_ particles in TiO_2_ pores, more precise tuning of the interfacial structure, a clearer understanding of the essential relationship between structure and ROS generation efficiency, and further optimization and acquisition of nanostructures with optimal performance. As a novel technology, however, there is no relevant report on using ALD technology in constructing nanosonosensitizer heterojunction and its application in tumor SDT.

**Scheme 1:**
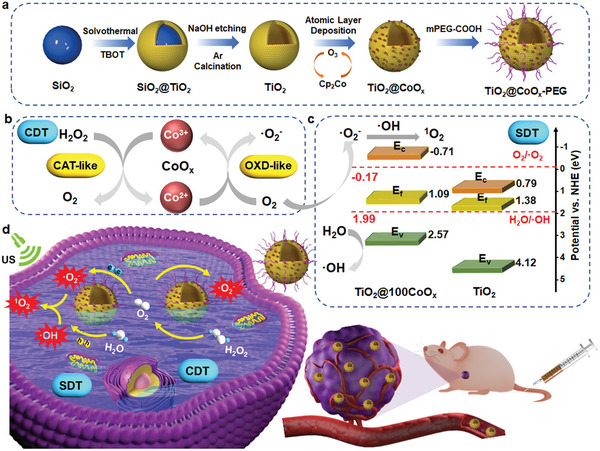
Schematic illustration. a) Construction of TiO_2_@CoO_x_, b) CAT‐like and OXD‐like activity mechanism of CoO_x_, and c) SDT enhancement mechanism TiO_2_@CoO_x_ nanoparticle. d) Schematic illustration of the synergistic SDT/CDT treatment.

Based on the aforementioned considerations, we proposed and realized a novel strategy to develop TiO_2_@CoO_x_ nanoparticles by ALD of CoO_x_ in TiO_2_ pores to achieve a high‐performance self‐oxygenated synergistic SDT/CDT against tumors (**Scheme** **1**). Due to the benefits of ALD, the loading amount and distribution of CoO_x_ in the TiO_2_ pores can be precisely controlled to maximize carrier separation efficiency and improve SDT performance. In addition, the energy band structure of the TiO_2_@CoO_x_ nanoparticles was effectively tuned so that the reactions on CB and VB could occur bilaterally, facilitating ROS production during SDT. In addition, CoO_x_ exhibited a cascade of H_2_O_2_→O_2_→·O_2_^−^ in response to the tumor microenvironment, which not only mitigated hypoxia during the SDT process but also caused chemodynamic cell damage. This paradigm enables the use of ALD‐prepared nanomedicines in catalytic tumor therapy and additional pharmaceutical applications.

## 2 Results and Discussion

### 2.1 Synthesis and Structural Characterizations

**Figure 1:**
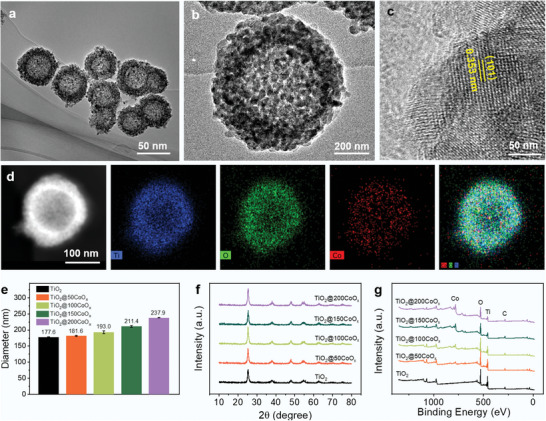
Evaluation of structural properties. a) Low‐ and b,c) high‐magnification TEM images of TiO_2_@100CoO_x_. d) EDX element mapping of TiO_2_@100CoO_x_. e) Particle size, f) XRD patterns, and g) XPS spectra of TiO_2_, TiO_2_@50CoO_x_, TiO_2_@100CoO_x_, TiO_2_@150CoO_x_, and TiO_2_@200CoO_x_.

Scheme 1a illustrates the fabrication of TiO_2_@CoO_x_ nanospheres by using SiO_2_ nanospheres (138 nm) as a template. First, SiO_2_@TiO_2_ nanospheres were formed by solvothermal coating SiO_2_ nanospheres with uniformly thick layers of TiO_2_. Then, monodisperse hollow porous crystalline TiO_2_ was obtained by etching the internal SiO_2_ with NaOH, followed by the Ar atmosphere calcination. CoO_x_ nanoparticles were deposited onto TiO_2_ spheres by ALD via alternately dosing cobaltocene (Cp_2_Co) and O_3_ into the chamber of a homemade ALD device. The as‐obtained nanocomposites were denoted as TiO_2_@*x*CoO_x_, where *x* represents the number of ALD cycles (Figure S1, Supporting Information). The ζ potentials (Figure S2, Supporting Information), UV–vis spectra (Figure S3, Supporting Information), and particle sizes (Figure S4, Supporting Information) of the intermediates as well as final nanoparticles were detected to observe their changes. The TEM image of crystallized TiO_2_ is shown in Figure S5a (Supporting Information). As depicted in the figure, crystallized TiO_2_ grains with an average grain size of 6.72 nm (Figure S5b, Supporting Information) comprise a TiO_2_ nanosphere with a particle size of ≈160 nm. After 100 cycles of CoO_x_ deposition, the contrast and size of TiO_2_@100CoO_x_ increased in comparison to pure TiO_2_ (**Figure** **1**a,b). High‐resolution transmission electron microscopy images of TiO_2_@100CoO_x_ reveal interplane distances of 0.353 nm (101) for anatase TiO_2_ (Figure 1c), and the Co elements were homogeneously dispersed in the porous TiO_2_ hollow spheres (Figure 1d). All data demonstrated the successful preparation of the TiO_2_@100CoO_x_ nanocomposite.

To optimize the heterojunction structure and achieve the best performance of the nanoparticles, 50, 150, and 200 cycles ALD of CoO_x_ were also performed on TiO_2_, and the effects of loading amount and interfacial structure on the carrier separation efficiency were analyzed further. The X‐ray fluorescence spectrometer (XRF) determined the percentage of CoO_x_ in TiO_2_@50CoO_x_, TiO_2_@100CoO_x_, TiO_2_@150CoO_x_, and TiO_2_@200CoO_x_ nanocomposites to be 6.35, 12.03 , 17.25 , and 21.18 wt.% (Table S1, Supporting Information). The nanocomposite sizes also increased with the CoO_x_ ALD cycles (Figure 1e). No characteristic CoO_x_ diffraction peaks were observed in the XRD patterns of TiO_2_@*x*CoO_x_, indicating that the CoO_x_ loaded by ALD are amorphous (Figure 1f). As depicted from X‐ray photoelectron spectroscopy (XPS) spectra, Ti peak intensities decreased while Co peak intensities increased with the deposition of CoO_x_ (Figure 1g). All data demonstrate that ALD allows for gradient tuning of nanomaterial structure and composition, which is important for performance optimization and understanding of their structure‐activity relationships.

### 2.2 ROS Generation Properties

To evaluate the SDT and CDT properties of the nanocomposites, the ROS generation capacity of each nanocomposite was assessed using corresponding chemical probes.

The 1,3‐diphenylisobednzofuran (DPBF) probe was used to examine the effect of CoO_x_ deposition cycles on SDT performance.

**Figure 2:**
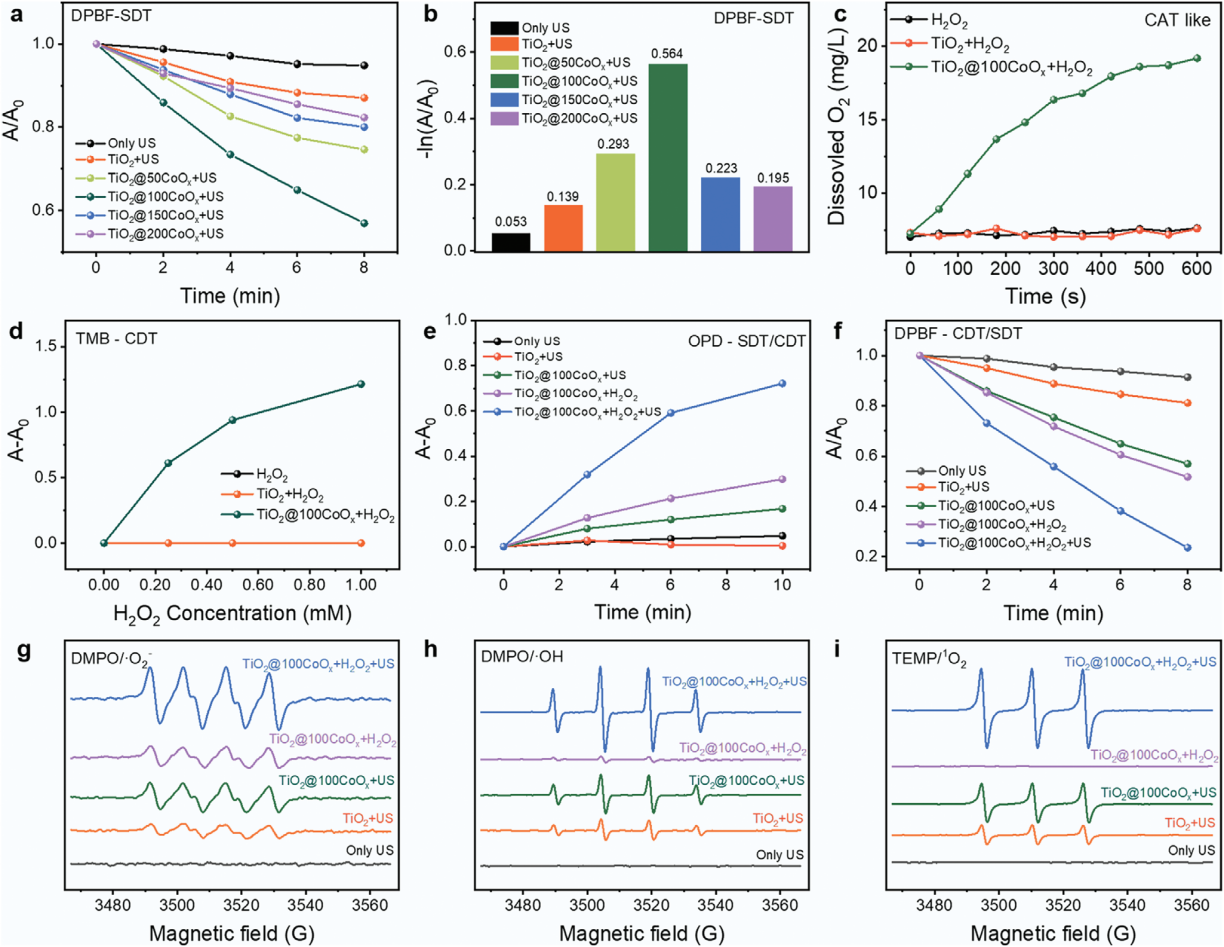
ROS generation through SDT and CDT processes. a) DPBF absorption intensities by US irradiation for different durations. b) DPBF oxidation efficiency for different nanocomposites under US irradiation. c) The O_2_ concentration changes in the H_2_O_2_ solution after the addition of TiO_2_ or TiO_2_@100CoO_x_. d) The generation of ROS in the CDT process using TMB as the probe. Production of ROS in the SDT and CDT process using e) OPD and f) DPBF as the probe. Electron spin resonance (ESR) spectra of g)·O_2_^−^ and h) ·OH generation using the spin trap DMPO and i) ^1^O_2_ using the spin trap TEMP under different processes.

All samples produced ROS under the US triggering, resulting in a gradual decrease in the characteristic absorption peak of DPBF (416 nm) with increasing US irradiation time (**Figure** **2**a; Figure S6, Supporting Information) due to the oxidation of DPBF to colorless 1,2‐dibenzoylbenzene (DBB). After the deposition of CoO_x_ on TiO_2_, the DPBF oxidation rate of TiO_2_@50CoO_x_ and TiO_2_@100CoO_x_ increased gradually, while those of TiO_2_@150CoO_x_ and TiO_2_@200CoO_x_ were gradually decreased, indicating that TiO_2_@100CoO_x_ possessed the highest sonocatalytic activity, which is 4.06 times that of pure TiO_2_ (Figure 2b). The DPBF oxidation kinetic curves and equations for TiO_2_@*x*CoO_x_ have been determined in Figure S7 and Table S2 (Supporting Information).

CoO_x_ is able to cascade H_2_O_2_ to produce O_2_ and then ·O_2_^−^, resulting in efficient CDT.^[^ ^17^ ^]^ There was a negligible difference in dissolved O_2_ between the TiO_2_ + H_2_O_2_ and H_2_O_2_ groups, but the concentration of dissolved O_2_ in the TiO_2_@100CoO_x_ + H_2_O_2_ group increased significantly (Figure 2c), confirming that CoO_x_ can decompose H_2_O_2_ into O_2_, which is consistent with the results of cytofluorometric imaging on HeLa cells (Figure S8, Supporting Information). 3, 3′, 5, 5′‐tetramethylbenzidine (TMB) was used as a probe to detect the CoO_x_‐induced ROS production in the presence of H_2_O_2_ at pH 6.0 (Figure 2d). ROS generation is negligible for TiO_2_ when H_2_O_2_ is added, whereas it gradually increases for TiO_2_@100CoO_x_ as the H_2_O_2_ concentration is increased. As the CoO_x_ ALD cycle increased, the generation of ROS also gradually increased (Figure S9, Supporting Information). Additionally, the oxidation of the chromogenic substrate *o‐*phenylenediamine (OPD) and DPBF were used to detect the synergetic production of ROS in the SDT and CDT processes (Figure 2e,f). Adding H_2_O_2_ to the system significantly enhanced US‐triggered ROS generation by the TiO_2_@100CoO_x_ (Figures S10 and S11, Supporting Information), demonstrating the synergetic effect of CDT and SDT performance.

Electron spin resonance (ESR) spectroscopy was utilized to further characterize the ROS yields. As depicted in Figure 2g–i, “TiO_2_ + US” generated ESR signals with intensity ratios of 1:1:1:1, 1:2:2:1, and 1:1:1 confirming the formation of DMPO/·O_2_^−^, DMPO/·OH, and TEMP/^1^O_2_ adducts, where ·O_2_^−^ and ·OH were generated on CB and VB and ^1^O_2_ was produced by disproportionation of ·O_2_^−^ and ·OH. In contrast, “TiO_2_@100CoO_x_ + US” produced significantly stronger ESR signal intensities compared to TiO_2_. Notably, for “TiO_2_@100CoO_x_ + H_2_O_2_”, the signal of DMPO/·O_2_^−^ was much more pronounced than that of DMPO/·OH and TEMP/^1^O_2_. As shown in “TiO_2_@100CoO_x_ + H_2_O_2_ + US”, adding H_2_O_2_ to the system further improves the DMPO/·O_2_^−^, DMPO/·OH and TEMP/^1^O_2_ ESR signals.

The results validate SDT performance enhancement and the synergy between the CDT and SDT functions of the nanocomposite. The gradient loading of CoO_x_ by ALD optimizes SDT performance due to the delicate formation of the heterostructure. CoO_x_ exhibited a cascade of H_2_O_2_→O_2_→·O_2_^−^ in response to the tumor microenvironment, which not only mitigated hypoxia during the SDT process but also gave the nanocomposites a CDT function. ESR results confirmed that the CDT function of CoO_x_ was primarily contributed by the formation of ·O_2_^−^. It also proved that the SDT performance was an H_2_O_2_‐sensitive procedure. The O_2_ produced from H_2_O_2_ increased the concentration of reactant on CB, further enhanced the SDT process and confirmed the synergistic effect of SDT and CDT.

### 2.3 SDT Performance Enhancement Mechanism

#### 2.3.1 Carrier Separation Efficiency Improvement

XPS was utilized to characterize the interaction between TiO_2_ and CoO_x_ for clarifying the effect of CoO_x_ deposition on TiO_2_ on SDT performance.

**Figure 3:**
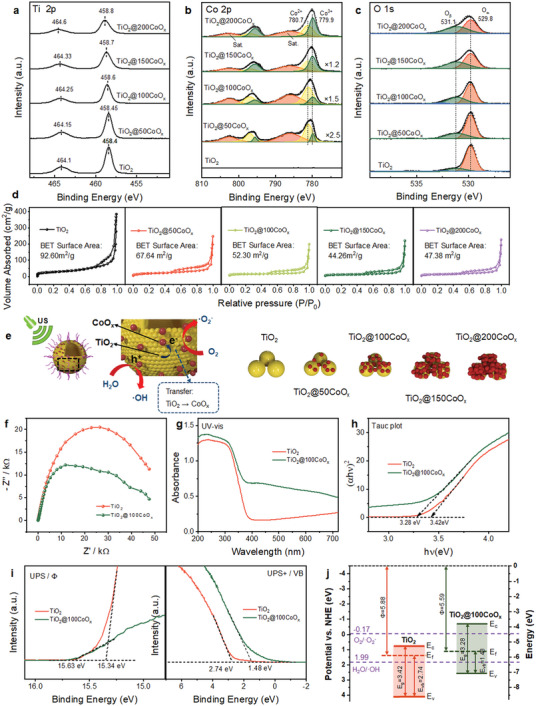
Catalytic mechanism analysis. a) Ti 2p b) Co 2p, and c) O 1 s X‐ray photoelectron spectroscopy (XPS) high‐resolution spectra of TiO_2_ and TiO_2_@CoO_x_. d) N_2_ adsorption−desorption isotherms of TiO_2_ and TiO_2_@CoO_x_. e) Mechanism for the enhanced SDT activity of TiO_2_@100CoO_x_. f) Electrochemical independent spectroscopy (EIS) Nyquist plots of TiO_2_ and TiO_2_@100CoO_x_. g) Diffuse reflectance spectroscopy (DRS) and the corresponding. h) Tauc plots of the TiO_2_ and TiO_2_@100CoO_x_. i) Ultraviolet photoelectron spectroscopy (UPS) of TiO_2_ and TiO_2_@100CoO_x_ for their wave function and valance band. j) Band structure of the TiO_2_ and TiO_2_@100CoO_x_ samples.

As the cycle number increased, the Ti peak gradually shifted to higher binding energies, which is evidence of electron transfer from TiO_2_ to CoO_x_ and confirmed the strong interaction between CoO_x_ and TiO_2_ (**Figure** **3**a).^[^ ^20^ ^]^ Consequently, it is likely that electrons can readily accumulate on the surface of CoO_x_ to contribute to the production of ·O_2_^−^, whereas holes remaining in TiO_2_ result in the production of ·OH (Figure 3e). The proportion of Co^3+^ increases as the number of deposition cycles increases, which is consistent with previous ALD deposition of CoO_x_ (Figure 3b).^[^ ^21^ ^]^ The increased cycle numbers resulted in a gradual increase in oxygen vacancies (O_α_) than lattice oxygen (O_β_) in the samples, which once again favored the sonocatalytic activity (Figure 3c).

On the other hand, Brunauer Emmett Teller (BET) analysis showed that specific surface areas of the samples decreased gradually with CoO_x_ deposition until cycle number 150, but increased slightly when cycle number up to 200. From the BET results (Figure 3d), as CoO_x_ was deposited, small CoO_x_ particles gradually filled the interstices between large TiO_2_ particles, and the CoO_x_ particle's density gradually increased so as to facilitate TiO_2_–CoO_x_ interaction, thereby gradually enhancing the sonocatalytic properties of TiO_2_@50CoO_x_ and TiO_2_@100CoO_x_. However, as additional CoO_x_ was deposited, excessive CoO_x_ depositions stacked upon one another because the TiO_2_ surface was already occupied by CoO_x_. Therefore, further deposition of CoO_x_ is not only useless for performance enhancement, but also impedes the migration of electrons from the TiO_2_–CoO_x_ interface to the nanocomposite surface for further reaction. Consequently, the performance of TiO_2_@150CoO_x_ begins to decline. TiO_2_@150CoO_x_ exhibited the smallest specific surface area, indicating that the TiO_2_ interstices had just been filled. Further deposition of CoO_x_ generated new specific surface areas, so the specific surface area of TiO_2_@200CoO_x_ is greater than that of TiO_2_@150CoO_x_, and its sonocatalytic properties were further diminished. Accordingly, the XPS and BET results explain the superior sonocatalytic performance of TiO_2_@100CoO_x_ (Figure 3e).

#### 2.3.2 Energy Band Structure Adaptation

Subsequently, the charge transfer properties of the samples were also measured using electrochemical independent spectroscopy (EIS). The decrease in the impedance arc radius of TiO_2_@100CoO_x_ relative to TiO_2_ suggests that the deposition of CoO_x_ decreases electronic impedance and increases carrier density and electron diffusion (Figure 3f), thereby favoring subsequent electron and hole reactions and SDT performance.^[^ ^22^ ^]^

The energy band structures of TiO_2_ and TiO_2_@100CoO_x_ were analyzed because the energy band structures of nanoparticles were closely related to the carrier separation efficiency, which significantly impacted their sonocatalytic performance. The UV–vis diffuse reflection spectrum (DRS) of TiO_2_ and TiO_2_@100CoO_x_ are illustrated in Figure 3g. The TiO_2_ absorption edge at ≈390 nm, demonstrates a weak photo response to visible light. Deposition of CoO_x_ on TiO_2_ resulted in enhanced absorption in the visible range, and the greater the number of deposition periods, the stronger the visible light absorption (Figure S12a, Supporting Information). The band gap of the catalyst can be evaluated using the formula: (*αhν*) = *A*(*hν* – *Eg*)^1/2^, and the band gaps of TiO_2_ and TiO_2_@100CoO_x_ were found to be 3.42 and 3.28 eV, respectively.^[^ ^23^ ^]^ As the number of ALD cycles increases, the band gap of the nanoparticles also decreases (Figure S12b, Supporting Information), demonstrating that the creation of heterojunctions facilitated the sonocatalytic reaction.

Ultraviolet photoelectron spectroscopy (UPS) measurements were used to investigate the effect of CoO_x_ on the valence band structure (VB) and work function (Φ) of TiO_2_. The Φ value represents the difference between the vacuum energy (0 eV) and the Fermi level energy (E_f_).^[^ ^24^ ^]^ Accordingly, a He UV light with an energy of 21.22 eV was utilized to excite the sample surface. The calculated Φ values for TiO_2_ and TiO_2_@100CoO_x_, respectively, were 5.88 and 5.59 eV (Figure 3h). The valence band maxima (VBM) of TiO_2_ and TiO_2_@100CoO_x_ were 2.74 and 1.48 eV, respectively, which corresponds to the difference between their E_f_ energy levels and valence band maxima (Figure 3i).

Combining DRS and UPS results, the energy band structures of TiO_2_ and TiO_2_@100CoO_x_ were determined, including the positions of the E_f_, VB, and CB (Figure 3j). The CB position of TiO_2_ was ˂(−0.17 V vs NHE for the potential of O_2_/·O_2_^−^), which explains why TiO_2_ can only generate ·OH when triggered by the US. The CB position of TiO_2_@100CoO_x_ was elevated, making its energy band structure match well with reaction energy on both sides, and bilateral reactions could be carried out smoothly.

### 2.4 Cellular Uptake and Cytotoxicity In Vitro

**Figure 4:**
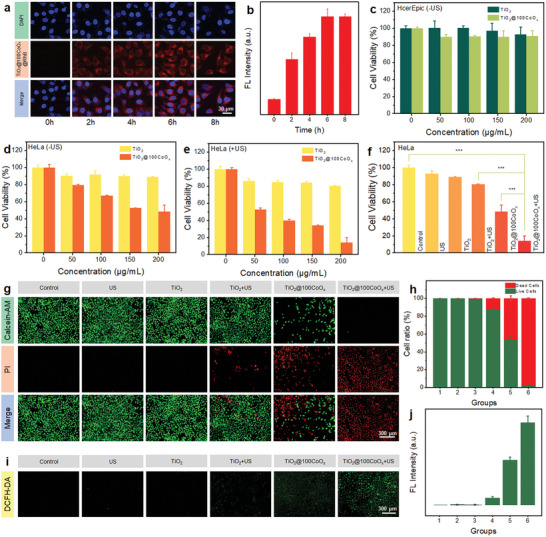
In vitro anti‐tumor efficiency by synergistic SDT/CDT. a) Time‐dependent cellular uptake of TiO_2_@100CoO_x_ and b) corresponding NIR fluorescence intensity. c) Relative cell viability of Hcerepic cells after incubation with TiO_2_ and TiO_2_@100CoO_x_ for 24 h at different nanoparticle concentrations. Relative cell viability of HeLa cells after incubating with TiO_2_ and TiO_2_@100CoO_x_ for 24 h at different nanoparticle concentrations: d) without US irradiation and e) with US irradiation. f) Relative cell viability of HeLa cells after different treatments (^***^ *p* <0.001). g,i) Live/dead cell staining and ROS images of HeLa cells after different treatments and h,j) corresponding NIR fluorescence intensity.

The uptake of TiO_2_@100CoO_x_ by HeLa cells was observed by labeling Rhodamine B (RhB) (**Figure** **4**a). The intracellular fluorescence intensity increased gradually with increased incubation time and reached a plateau at 6 h (Figure 4b), indicating that the TiO_2_@100CoO_x_ was efficiently taken up by the HeLa cells.

Standard 3‐(4,5‐dimethylthiazol‐2‐yl)−2,5‐diphenyltetrazolium bromide (MTT) assays were utilized to assess the biocompatibility of TiO_2_ and TiO_2_@100CoO_x_ on cervical epithelial cells (HcerEpic), as well as its SDT/CDT synergistic effect on HeLa cells. Before conducting biological experiments, both nanoparticles were modified with methoxy‐polyethylene glycol‐carboxyl‐2000 (mPEG‐COOH) to enhance their water solubility, stability, and biocompatibility. The modification of mPEG‐COOH was verified by Fourier transform infrared (FT‐IR) spectra and thermogravimetric analysis (TGA). Figure S13a (Supporting Information) showed two additional characteristic peaks at 1108 and 951 cm^−1^, which corresponded to the C─O─C group of mPEG─COOH, indicating that mPEG─COOH had chemical bond formation with TiO_2_. TGA spectra (Figure S13b, Supporting Information) also proved and the surface modification was successful. Time‐dependent particle size of colloidal TiO_2_@100CoO_x_‐PEG (Figure S14, Supporting Information) demonstrating its stability in PBS and water. “TiO_2_‐PEG” and “TiO_2_@100CoO_x_‐PEG” are abbreviated to “TiO_2_” and “TiO_2_@100CoO_x_” respectively in cellular and in vivo experiments. Without US irradiation, TiO_2_ and TiO_2_@100CoO_x_ demonstrated good cytocompatibility and were not cytotoxic to HcerEpic cells, quantitative cell viability exceeded 90% at concentrations as high as 200 µg mL^−1^ were observed (Figure 4c). After US irradiation, TiO_2_ was virtually non‐cytotoxic to HcerEpic cells, as cell viability remained >90%, demonstrating the low ROS production efficiency of pristine TiO_2_ under US irradiation. Comparatively, the viability of HcerEpic incubated with TiO_2_@100CoO_x_ after US irradiation (1.0 MHz, 1.0 W cm^−2^, 50% duty cycle) was 57%, confirming that the ALD of CoO_x_ on TiO_2_ successfully constructed heterojunctions and enhanced ROS generation under US (Figure S15, Supporting Information). As for the lethality of HeLa cells, TiO_2_ was also non‐toxic to HeLa cells, whereas TiO_2_@100CoO_x_ showed a significant injurious ability to HeLa cells, which was attributed to the CDT effect of CoO_x_ on tumor cells in the presence of high concentrations of endogenous H_2_O_2_ (Figure 4d). Survival of HeLa cells treated with TiO_2_@100CoO_x_ (14%) was significantly lower than that of cells treated with TiO_2_ (78%) after US irradiation, further confirming the enhanced effect of heterojunctions on SDT and the SDT/CDT synergistic effect on tumor cells (Figure 4e). Significant differences between groups are shown in Figure 4f.

The superior SDT/CDT performance of TiO_2_@100CoO_x_ was also validated by live/dead co‐staining experiments (Figure 4g). The fluorescence statistics of live and dead cells were in general agreement with the MTT (Figure 4h). To determine the increase in intracellular ROS levels 2′, 7′‐dichlorofluorescein diacetate (DCFH‐DA) staining was performed (Figure 4i). No obvious ROS fluorescence signal was observed in HeLa cells in the “control”, “US”, and “TiO_2_” groups. For the “TiO_2_ + US” group, a very weak ROS fluorescence signal was observed, indicating the poor sonocatalytic performance of pristine TiO_2_. The CDT effect generated ROS, which resulted in a stronger fluorescence signal in the “TiO_2_@100CoO_x_” group. The “TiO_2_@100CoO_x_ + US” group exhibited a significantly brighter ROS fluorescence signal than the sum of the “TiO_2_ + US” and “TiO_2_@100CoO_x_” groups, confirming that the heterojunction increased intracellular ROS production. The ROS fluorescence intensity data (Figure 4j), in conjunction with MTT survival and AM/PI, demonstrated the excellent SDT/CDT synergistic anti‐tumor effect of TiO_2_@100CoO_x_ in vitro.

### 2.5 In Vivo Biodistribution and Synergistic Effect of SDT and CDT

**Figure 5:**
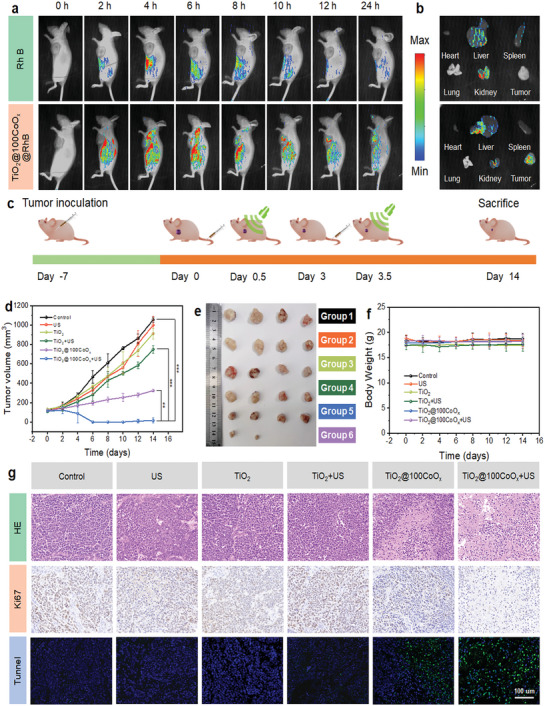
In vivo biodistribution and SDT/CDT synergistic anti‐tumor analysis. a) In vivo fluorescence images of mice injected I.V. with free RhB and TiO_2_@100CoO_x_@RhB, respectively. b) Fluorescence images of heart, liver, spleen, lungs, kidneys, and tumor tissues of mice 24 h after I.V. injection of RhB and TiO_2_@100CoO_x_@RhB. c) Therapeutic protocol on HeLa tumor‐bearing model. d) Tumor growth curves and e) pictures of tumors after the six treatment groups. (*n* = 4, ^**^ *p* <0.01, ^***^ *p* <0.001) (1, control; 2, US alone; 3, TiO_2_ alone; 4, TiO_2_ + US; 5, TiO_2_@100CoO_x_; and 6, TiO_2_@100CoO_x_+ US). f) Body weight curves of mice in different treatment groups. g) H&E, TUNEL, and Ki67 staining of tumor tissues of each group.

Given TiO_2_@100CoO_x_’s superior in vitro anti‐tumor efficacy, its biological behaviours in vivo were systematically evaluated. The results showed that RhB‐labeled TiO_2_@100CoO_x_ could accumulate rapidly in the tumor region and reached the maximum accumulation at 10 h post‐injection, and TiO_2_@100CoO_x_ still had a high tumor retention rate at 24 h post‐injection compared with free RhB, which had almost no retention in the tumor region (**Figure** **5**a). The mice were sacrificed 24 h after I.V. injection, and it was found that RhB‐labeled TiO_2_@100CoO_x_ accumulated mainly in the tumor tissues, but not in other major organs, including the heart, liver, spleen, lungs, and kidneys (Figure 5b).

Healthy mice were used to evaluate the biocompatibility of TiO_2_ and TiO_2_@100CoO_x_ in vivo. Hematoxylin and eosin (H&E) staining of major organs after exposure to different groups revealed no significant differences between groups, indicating that TiO_2_ and TiO_2_@100CoO_x_ are biocompatible and safe (Figure S16, Supporting Information). The HeLa‐bearing BALB/c mice were subjected to a treatment protocol consisting of a dose of 5.0 mg k^−1^g and US irradiation (1.0 MHz, 50% duty cycle, 1.0 W cm^−2^, 5 min). HeLa tumor‐bearing mice were randomly divided into six treatment groups: 1) control; 2) US alone; 3) TiO_2_ alone; 4) TiO_2_ + US; 5) TiO_2_@100CoO_x_; and 6) TiO_2_@100CoO_x_ + US. US irradiation was performed 10 h after intravenous injection on day 0 and day 3, respectively (Figure 5c). During the 14‐day period, the tumor sizes and body weights were recorded every 2 days. As shown in Figure 5d, in the absence of US irradiation, TiO_2_‐induced inhibition of tumor growth was 18% (Figure S17, Supporting Information). In contrast, the tumor inhibition in the TiO_2_@100CoO_x_ group was 72%, indicating that CoO_x_ has a significant CDT effect. However, the tumor growth inhibition by “TiO_2_ + US” remained limited (33%). In contrast, the “TiO_2_@100CoO_x_ + US” group demonstrated the most pronounced inhibition of tumor growth (94%), indicating that heterojunctions were more effective than single components and confirming the synergistic effect of SDT and CDT. The above results are consistent with the photos of the HeLa tumor‐bearing mice (Figure S18, Supporting Information) and the tumor images (Figure 5e), as well as the weight results of mice after being sacrificed on day 14 (Figure S19, Supporting Information). In addition, the weight monitoring results showed no significant changes in the body weight of tumor‐bearing mice during treatment, further demonstrating the biosafety of the nanoparticles and treatment regimen (Figure 5f).

The tumor tissues were also evaluated for necrosis and apoptosis using H&E staining, terminal deoxynucleotidyl transferase‐mediated dUTP‐biotin nick end labelling (TUNEL) staining, and immunohistochemical (Ki‐67) staining. The results demonstrated that the “TiO_2_@100CoO_x_ + US” (SDT + CDT) group exhibited the most pronounced necrosis and apoptosis in comparison to the “TiO_2_@100CoO_x_” (CDT) or “TiO_2_ + US” (unmodified SDT) groups, confirming the efficient synergistic effect of SDT and CDT of TiO_2_@100CoO_x_ nanocomposite (Figure 5g).

### 2.6 SDT and CDT Combination Induces Transcriptome Alterations

**Figure 6:**
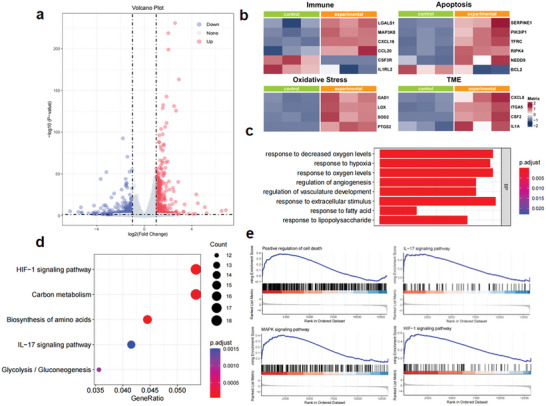
RNA sequencing analysis of HeLa cells of the control group and the SDT/CDT treatment group. a) Volcano map (*p*‐adjust <0.05). b) Heatmaps of DEGs associated with immune, apoptosis, oxidative stress, and TME. c) KEGG and d) GO enrichment analyses of functions and signal pathways that the related DEGs participated in. e) GSEA enrichment plots of signal pathways in the SDT/CDT treatment group compared to the control group.

RNA sequencing was used to analyze differences in gene expression in HeLa cells before and after TiO_2_@100CoO_x_ and US treatment by cluster analysis and enrichment analysis. The volcano plot (**Figure** **6**a) revealed that 362 genes were significantly up‐regulated and 382 were significantly down‐regulated (*p* <0.05). Additionally, cluster analysis was used to screen up‐ and down‐regulated HeLa gene expression (Figure S20, Supporting Information). As shown in Figure 6b, the expression of genes associated with immunity, apoptosis, oxidative stress, and TME differed significantly between the SDT/CDT group and the control group. TiO_2_@100CoO_x_‐mediated SDT/CDT synergistic treatment may regulate immune chemokines (e.g., CXCL16, CCL20, etc.), immune responses (e.g., IL1RL2, CSF3R, MAP3K8, etc.), and cell adhesion (e.g., SERPINE1, TFRC, etc.). In addition, a number of immune‐related proteins, such as LGALS1, IRF5, and IL‐7, were significantly up‐regulated in the experimental group, indicating that the treatment may induce the immune system to recruit a large number of immune cells into TME. In the experimental group, the expression of apoptosis‐related genes such as PIK3IP1 was significantly up‐regulated, which induced apoptosis. Moreover, the expression of genes associated with oxidative stress, such as LOX, GAD1, SOD2, and PTGS2, was significantly up‐regulated, indicating that ROS generated during SDT/CDT disrupted the intracellular redox balance and activated the intracellular oxidative stress system.

Additionally, gene ontology (GO) enrichment analysis demonstrated that DEGs were primarily focused on cellular hypoxia, intracellular angiogenesis, cell proliferation, and protein phosphorylation (Figure 6c), which are all integral to the development of tumors. Kyoto Encyclopedia of Genes and Genomes (KEGG) enrichment results indicated that DEGs are primarily involved in MicroRNAs, carbon metabolism, the IL‐17 signalling pathway, and the MAPK signalling pathway in cancer (Figure 6d). The enrichment of the HIF‐1 signalling pathway demonstrates directly that the treatment affects transcriptional regulatory processes associated with hypoxia in cells, which may play a significant role in the treatment of cancer. In addition, the enrichment of the IL‐17 signalling pathway indicates that TiO_2_@100CoO_x_ + US promoted the death of tumor cells by modulating the immune response. To further comprehend the therapeutic mechanism (Figure 6e), gene set enrichment analysis (GSEA) was performed. The synergistic treatment of SDT and CDT increased the expression of genes involved in the positive regulation of cell death, the IL‐17 signalling pathway, the MAPK signalling pathway, and the HIF‐1 signalling pathway. The transcriptome analysis provides the foundation for a comprehensive investigation of the anti‐tumor mechanism of TiO_2_@100CoO_x_.

## 3 Conclusion

An optimized nanoplatform with synergistic CDT/SDT effects was successfully built via ALD technology for the effective treatment of cancer. Accordingly, we constructed a multifunctional TiO_2_@CoO_x_ heterojunction using ALD technology and optimized the SDT performance by precisely tuning its interface and valance band structure. The CoO_x_ was found to possess a cascade of H_2_O_2_→O_2_→·O_2_^−^ in response to the tumor microenvironment, which not only enhances SDT performance further but also demonstrates a CDT effect. Thus, the current study represents a potential paradigm for the use of advanced ALD technology to prepare nanocomposites to improve tumor‐catalyzed therapies, and it may also serve as a resource for future research on related pharmaceutical applications.

## Conflict of Interest

The authors declare no conflict of interest.

## Author Contributions

W.L. and R.S. contributed equally to this work. W.L. conceptualized the study, data analyses and wrote the manuscript. R.S. performed the experiments and data analyses. L.L., B.W., J.M., and L.G. contributed to the experimental design and performed the analysis with constructive discussions. H.W. and S M. assisted with material characterization and testing. C.Z. and B.Z. conceived the idea and supervised the work. H.D., Y.Q., and L.Y. acquired funding and supervised. All authors revised the manuscript and approved the final version.

## Supporting information

Supporting Information

## Data Availability

The data that support the findings of this study are available from the corresponding author upon reasonable request.

## References

[1] X. Li , J. Kim , J. Yoon , X. Chen , Adv. Mater. 2017, 29, 16068. 10.1002/adma.201606857 PMC5544499 28370546

[2] Z. Wang , B. Liu , Q. Sun , L. Feng , F. He , P. Yang , S. Gai , Z. Quan , J. Lin , ACS Nano 2021, 15, 12342. 34160201 10.1021/acsnano.1c04280

[3] T. Inui , K. Makita , H. Miura , A. Matsuda , N. Sakanoto , Anticancer Res. 2014, 34, 4589. 25075104

[4] T. Inui , H. Amitani , K. Kubo , D. Kuchiike , Y. Uto , T. Nishikata , M. Mette , Anticancer Res. 2016, 36, 3767. 27354652

[5] Y. Ji , W. Che , Chin. J. Oncol. 2019, 41, 638. 10.3760/cma.j.issn.0253-3766.2019.08.015 31434458

[6] X. Xing , S. Zhao , T. Xu , L. Huang , Y. Zhang , M. Lan , C. Lin , X. Zheng , P. Wang , Coordin. Chem. Rev. 2021, 445, 214087.

[7] a) F. Gong Fei , L. Cheng , N. Yang , O. Betzer , L. Feng , Q. Zhou , Y. Li , R. Chen , R. Popovtzer , Z. Liu , Adv. Mater. 2019, 23, 1900730; 10.1002/adma.201900730 30977247

[8] a) L. Parizot , T. Chave , M. E. Galvez , H. Dutilleul , P. D. Costa , S. I. Nikitenko , Appl. Cata. B 2019, 241, 570; 10.1016/j.ultsonch.2020.105270 32736303

[9] a) Y. Wang , D. Zhao , W. Ma , C. Chen , J. Zhao , Environ. Sci. Techno. 2008, 42, 6173; 10.1021/es800168k 18767683

[10] X. Wang , X. Zhong , L. Bai , J. Xu , F. Gong , Z. Dong , Z. Yang , Z. Zeng , Z. Liu , L. Cheng , J. Am. Chem. Soc. 2020, 142, 6527. 32191455 10.1021/jacs.9b10228

[11] a) S. Bai , N. Yang , X. Wang , F. Gong , Z. Dong , Y. Gong , Z. Liu , L. Cheng , ACS Nano 2020, 14, 15119; 33185089 10.1021/acsnano.0c05235

[12] Y. Cao , T. Wu , W. Dai , H. Dong , X. Zhang , Chem. Mater. 2019, 31, 9105.

[13] a) Y. Zhao , J. Liu , M. He , Q. Dong , L. Zhang , Z. Xu , Y. Kang , P. Xue , ACS Nano 2022, 16, 12118; 35904186 10.1021/acsnano.2c02540

[14] W. Xu , C. Dong , H. Hu , X. Qian , L. Chang , Q. Jiang , L. Yu , Y. Chen , J. Zhou , Adv. Funct. Mater. 2021, 31, 2103134.

[15] Z. Cao , G. Yuan , L. Zeng , L. Bai , X. Liu , M. Wu , R. Sun , Z. Chen , Y. Jiang , Q. Gao , Y. Chen , Y. Zhang , Y. Pan , J. Wang , ACS Nano 2022, 16, 10608. 35759554 10.1021/acsnano.2c02177

[16] a) Y. Wang , C. Zhu , G. Zuo , Y. Guo , W. Xiao , Y. Dai , J. Kong , X. Xu , Y. Zhou , A. Xie , C. Sun , Q. Xian , Appl. Cata. B 2020, 278, 119298;

[17] a) M. Sun , S. Huang , G. Su , X. Wang , Z. Lu , Y. Wang , T. Liu , Y. Jiang , C. Song , H. Rao , Chem. Eng. J. 2022, 437, 134414;

[18] K. Koga , ACS Appl. Mater. Interfaces 2020, 12, 20806. 32212617 10.1021/acsami.9b23290

[19] b) B. J. O'Neill , D. H. K. Jackson , J. Lee , C. Canlas , P. C. Stair , C. L. Marshall , J. W. Elam , T. F. Kuech , J. A. Dumesic , G. W. Huber , ACS Catal. 2015, 5, 1804;

[20] a) Z. Tian , Y. Da , M. Wang , X. Dou , X. Cui , J. Chen , R. Jiang , S. Xi , B. Cui , Y. Luo , H. Yang , Y. Long , Y. Xiao , W. Chen , Nat. Commun. 2023, 14, 142; 36627303 10.1038/s41467-023-35875-9 PMC9831984

[21] a) G. Capilli , Y. Chen , T. Szkopek , M. Cerruti , ACS Nano 2022, 16,12488; 35921169 10.1021/acsnano.2c03877

[22] a) B. Chu , S. Liu , Q. Qin , R. Zhao , K. Chen , X. Hou , R. Li , C. Li , J. Chen , L. Dong , B. Li , Adv. Funct. Mater. 2023, 33, 2212448;

[23] C. Wang , Y. Zhao , T. Ma , Y. An , R. He , J. Zhu , C. Chen , S. Ren , F. Fu , D. Zhao , X. Li , Nat. Energy 2022, 7, 744.

[24] M. Holbrook , Y. Chen , H. Kim , L. Frammolino , M. Liu , C. Pan , M. Chou , C. Zhang , C. Shih , ACS Nano 2023, 17, 6966. 36946518 10.1021/acsnano.3c01082

