# Supplementary material for: Precise Design of TiO2@CoOx Heterostructure via Atomic Layer Deposition for Synergistic Sono‐Chemodynamic Oncotherapy
Source: Adv Sci (Weinh). 2024 Feb 4;11(14):2304046. doi: 10.1002/advs.202304046 (PMC11005734; doi:10.1002/advs.202304046)
Supplement: Supplementary file 1 — Supporting Information [file ADVS-11-2304046-s001.pdf]

ADVANCED SCIENCE
Open Access

Supporting Information

for *Adv. Sci.*, DOI 10.1002/advs.202304046

Precise Design of TiO_2@CoO_x Heterostructure via Atomic Layer Deposition for Synergistic Sono-Chemodynamic Oncotherapy

Wen Liu, Runrun Shao, Lingyun Guo, Jianliang Man, Chengwu Zhang, Lihong Li, Haojiang Wang, Bin Wang, Lixia Guo, Sufang Ma, Bin Zhang, Haipeng Diao*, Yong Qin* and Lili Yan**

Precise design of TiO_2@CoO_x heterostructure via atomic layer deposition for synergistic sono-chemodynamic oncotherapy

Wen Liu, Runrun Shao, Lingyun Guo, Jianliang Man, Chengwu Zhang, Lihong Li, Haojiang Wang, Bin Wang, Lixia Guo, Sufang Ma, Bin Zhang, Haipeng Diao,* Yong Qin,* and Lili Yan**

W. Liu, R. Shao, L. Guo, J. Man, C. Zhang, L. Li, H. Wang, B. Wang, S. Ma, H. Diao, L. Yan

Basic Medical College

Shanxi Medical University

Taiyuan 030001, P. R. China

E-mail addresses: diaohp@sxmu.edu.cn, yanlili@sxmu.edu.cn

W. Liu, H. Diao, L. Yan

Key Laboratory of Cellular Physiology at Shanxi Medical University

Ministry of Education

Taiyuan 030001, P. R. China

L. Guo

Pharmacy College

Shanxi Medical University

Taiyuan 030001, P. R. China

B. Zhang, Y. Qin

State Key Laboratory of Coal Conversion

Institute of Coal Chemistry

Chinese Academy of Sciences

Taiyuan 030001, P. R. China

E-mail addresses: qinyong@sxicc.ac.cn, zhangbin2009@sxicc.ac.cn

Materials: The ethanol and ammonium hydroxide were obtained from Damao chemical reagent factory. Tetraethyl orthosilicate (TEOS), tetrabutyl titanate (TBOT) and dimethyl sulfoxide (DMSO) were provided by Macklin. Rhodamine B (RhB) and 3,3',5,5'-tetramethylbenzidine (TMB) were provided by Aladdin. 3-(4,5)-dimethylthiahiazo (-z-y1)-3,5-di-phenytetrazoliumromide (MTT), calcein acetoxymethyl ester (Calcein-AM), propidium iodide (PI) and 2′,7′-dichlorofuorescin diacetate (DCFH-DA) were provided by Solarbio. mPEG-COOH was provided by Xi'an ruixi Biological Technology Co., Ltd. All chemicals were of analytical grade and used without further purification.

Synthesis of SiO_2 spheres: SiO_2 spheres were prepared using a modified Stöber method. The components TEOS (1.7 mL), deionised water (0.8 mL), ammonia solution (28 wt.%, 1.7 mL), and ethanol (40 mL) were combined. After vigorous stirring for 24 h, the SiO_2 sample was separated by centrifugation (12000 rpm for 10 min) and washed three times with ethanol and distilled water, respectively.

Synthesis SiO_2@TiO_2: About 0.15 g of SiO_2 was dispersed in 90 mL of ethanol and ultrasonically treated for 60 min. The SiO_2 suspension was then mixed with the ammonia solution (28 wt.%, 0.8 mL) and TBOT (1.5 mL). This suspension was transferred into a round-bottom flask and stirred for 24 h in a water bath at 45 °C. The sample was centrifuged and washed three times with ethanol and distilled water, respectively. The sample was dried at 70 °C for 12 h, before being calcined at 400 °C in air for 2 h. The resulting sample was designated as SiO_2@TiO_2.

Synthesis hollow TiO_2: The SiO_2@TiO_2 particles (0.1 g) were dispersed in

deionised water (20 mL) for 60 min using ultrasound. The resultant suspension was then treated with a NaOH solution (20 mL, 2 mol·L^{-1}), and the etching reaction was allowed to proceed for 12 h at 70 °C with stirring. The etched sample was isolated by centrifugation, and the centrifugal supernatant was collected for later use. The hollow TiO_2 was annealed in air at 400 °C to gain crystalised TiO_2.

Synthesis TiO_2@CoO_x: In a homemade ALD reactor, CoO_x was deposited onto TiO_2. Before ALD, the TiO_2 nanospheres were dispersed in ethanol and ultrasonically stirred for 20 min. The TiO_2 nanospheres were subsequently dripped onto a quartz wafer for air drying. After the samples were completely dried, they were transferred to the ALD reactor. To deposit CoO_x, $Co(cp)_2$ and O_3 pulses of varying cycle numbers were alternately introduced into the reactor. The reaction parameters for CoO_x deposition by ALD were as follows. The temperature of deposition was 220 °C. $Co(cp)_2$ and O_3 were the precursors utilised. Prior to being introduced into the reactor, the $Co(cp)_2$ precursor was heated to 80 °C in order to achieve an acceptable vapour pressure. The carrier gas consisted of 50 sccm of high-purity nitrogen. The pulse time for $Co(cp)_2$ was 5.5 s, the holding time was 16 s, and the N_2 blowing time was 25 s. The pulse time for O_3 was 0.10 s, the holding time was 12 s, and the blowing time for N_2 was 25 s. The samples after CoO_x ALD are labelled TiO_2@$x$$CoO_x$ for convenience, where x is the number of CoO_x deposition cycles.

Synthesis TiO_2*-PEG* and TiO_2*@100*CoO_x *-PEG:* TiO_2 or 10 mg of TiO_2@$x$$CoO_x$ was dissolved in water along with 100 mg of mPEG-COOH and stirred for 24 h. Then, TiO_2@$x$$CoO_x$-PEG was obtained by re-dispersing it in water after purification.

Sonodynamic performance: For $^1O_2$ or $\cdot OH$ detection, 0.1mM DPBF or 0.5 mM OPD was first added to TiO_2@*x*CoO_x solution (200 μg mL^{-1}), respectively. Subsequently, the DPBF and OPD absorption changes for the TiO_2@100CoO_x solution under US irradiation (1 MHz, 1.0 W cm^{-2}, 50% duty cycle) were measured to determine $^1O_2$ and $\cdot OH$ generation, respectively.

Chemodynamic performance: For the measurement of $\cdot OH$ generated by TiO_2@*x*CoO_x via CDT, TMB (0.8 mM) was added to a TiO_2@*x*CoO_x solution (200 μg mL^{-1}, 2 mL). The mixture was then incubated with various concentrations of H_2O_2 (0.25, 0.50, and 1.00 mM) in PBS (pH 5.5 or 6.5). After 2 min of incubation, UV–vis absorption spectra were acquired to determine the TMB absorbance at 650 nm.

Catalase-like activity performance: The dissolved O_2 generation from H_2O_2 (1mM) by TiO_2 and TiO_2@*x*CoO_x (200 μg mL^{-1}) were measured by a portable dissolved oxygen meter (JPSJ-606F Leici Instrument Co., Ltd., Shanghai, China).

ESR measurement: For ESR measurement of $\cdot O_2^-$ $\cdot OH$, and $^1O_2$, DMPO (200 mM) and TEMP (400 mM) and were added to the TiO_2@*x*CoO_x solution (0.2 mg mL^{-1}), respectively. Accordingly, an ESR spectrometer was used to detect $\cdot O_2^-$ $\cdot OH$, and $^1O_2$ generation by TiO_2@*x*CoO_x with or without US irradiation.

Cellular uptake: Rhodamine B complex (RhB) was loaded on TiO_2@100CoO_x in order to qualify for the ability of cell fluorescence imaging. Typically, 10 mg of Rhodamine B and 1 mg/mL of TiO_2@100CoO_x in 3 mL of methanol are thoroughly combined and stirred for 24 h in the dark. The TiO_2@100CoO_x - RhB was obtained by centrifuging and washing with PBS. Prior to use, TiO_2@100CoO_x - RhB was dispersed

in PBS. After HeLa cells were incubated with $TiO_2@100CoO_x$ - RhB (200 μg mL^{-1}) for various time periods (0 h, 2 h, 4 h, and 8 h), they were washed with PBS and incubated with DAPI (1×10^{-6} M) for 30 min. Subsequently, the cell fluorescence images (RhB: excitation: 488 nm; emission: 525 nm) were captured using a confocal fluorescence microscope.

Cytotoxicity Measurement and In Vitro CDT and SDT: Using HeLa and HcerEpic cells as models, the in vitro dark cytotoxicity of samples was evaluated using standard MTT assays. Briefly, a 96-well plate containing 8000 cells per well was seeded and incubated overnight. The cells were then treated with various concentrations of $TiO_2@xCoO_x$, ranging from 0 to 200 μg mL^{-1}. Subsequently, the cells were subjected to the following disposals, including (1) Control, (2) US, (3) TiO_2, (4) TiO_2+US, (5) $TiO_2@100CoO_x$, and (6) $TiO_2@100CoO_x$ +US. After co-incubating cells with $TiO_2@xCoO_x$ (200 μg mL^{-1}) for 24 h, the US treatments (0.8 W cm^2, 1.0 MHz, 50% duty cycle, 5 min) were performed. After 24 h of incubation in the dark, the medium containing the nanocomposites were removed, and 10 μL of a MTT solution (diluted in a culture medium to a final concentration of 0.5 mg mL^{-1}) was added to each well, which was then cultured for an additional 4 h. Finally, 150 μL of dimethyl sulfoxide (DMSO) were added to each well in place of the supernatant. The plate was shaken for 10 min and examined with a microplate reader at a wavelength of 490 nm. The Results were expressed as the percentage of cell viability. 1×10^6 HeLa cells were seeded into each well of a six-well plate in order to visualise living and dead cells. Following the aforementioned procedures, cells were stained with Calcein-AM and PI for 40 min,

washed with PBS and observed via inverted fluorescence microscopy.

Detection of Intracellular ROS: To detect ROS production, HeLa cells were seeded and cultured overnight in 6-well plates. After coincubation with $TiO_2@xCoO_x$ (200 μg mL^{-1}), discarding the medium and rinsing the cells, 1 x 10^{-6} M DCFH-DA was added to the wells for 40 min. Ten cells were gently washed twice and then subjected to US irradiation (1.0 MHz, 1.0 W·cm^{-2}, 5 min). The cells were immediately observed via inverted fluorescence microscopy. To detect cell uptake, HeLa cells were seeded into a Confocal Dish at a density of 5×10^4 and cultured for 24 h.

Tumour modelling: Female Balb/c mice were purchased from Beijing Vital River Laboratory Animal Technology Co., Ltd. as a model for mice carrying HeLa cells. All the experiments carried out on the mice were strictly in accordance with the institutional Animal Care and Use Committee of Shanxi Medical University (IACUC 2017-018).

In vivo biodistribution: To determine the biodistribution of nanomaterials, 200 μL RhB and $TiO_2@CoO_x@$Rh B dissolved in PBS were injected to tumor-bearing balb/c mice via tail vein. Using the multimodal Xtreme imaging system, the circulation process was monitored and fluorescence imaging (Ex/Em: 530/600 nm) was obtained at different time nodes (0, 2, 4, 6, 8, 10, 12 and 24 h). The mice were sacrificed 24 h after drugs administration for tissues and organs (heart, liver, spleen, lung, kidney, and tumor) fluorescence imaging."

In vivo biocompatibility assay: A total of 24 Balb/c female mice were divided into 6 groups (n = 4 per group): (1) Control, (2) US, (3) TiO_2, (4) TiO_2+US, (5) $TiO_2@100CoO_x$, (6) $TiO_2@100CoO_x$ +US. (5 mg/kg, 200 μL, i.v. injection) After

intravenous injection, the individual's body weight was measured every 2 days. The principal organs were stained with hematoxylin and eosin (H&E) following 14 days of feeding.

In vivo anti-tumour therapy: The HeLa tumor-bearing mice were divided into six groups (n = 4): (1) Control, (2) US, (3) TiO_2, (4) TiO_2+US, (5) TiO_2@100CoO_x, (6) TiO_2@100CoO_x+US. (20 mg kg^{-1}, 200 μL, i.v. injection). The tumour site of HeLa tumour-bearing mice was treated with US irradiation (1.0 MHz, 50% duty cycle, 1 W cm^{-2}, 5 min) 8 h after nanocomposites intravenous injection. The procedure was repeated twice, every other day. In addition, the tumor size and body weight were also recorded every other day. The mice were then euthanised, and their tumours were resected for H&E, TUNEL, and Ki-67 analyses. The tumour volume was calculated using Equation (1), and the tumour inhibition rate was determined using Equation (2).

$$\text{Tumor volume} = \frac{\text{length} \times \text{width}^2}{2} \quad (1)$$

$$\text{Tumor inhibition rate} = 1 - \frac{V_{\text{experiment}}}{V_{\text{control}}} \times 100\% \quad (2)$$

In Vitro RNA Sequencing: RNA was extracted from Hela cells in both the control and TiO_2@100CoO_x + US treated groups and sent to Suzhou PANOMIX Biomedical Tech Co., LTD for sequencing. Gene expression profiles were analysed using htseq-count and cufflinks. Subsequently, the list of differentially expressed genes generated by DESeq was analysed based on the GO and KEGG databases.

Statistical Analysis: All statistical analyses in this paper were presented as mean ± standard deviation (SD), and the significance between two groups of the data in this work was analyzed based on Student's two-tailed t-test (*p < 0.05, **p < 0.01, ***p <

0.001).

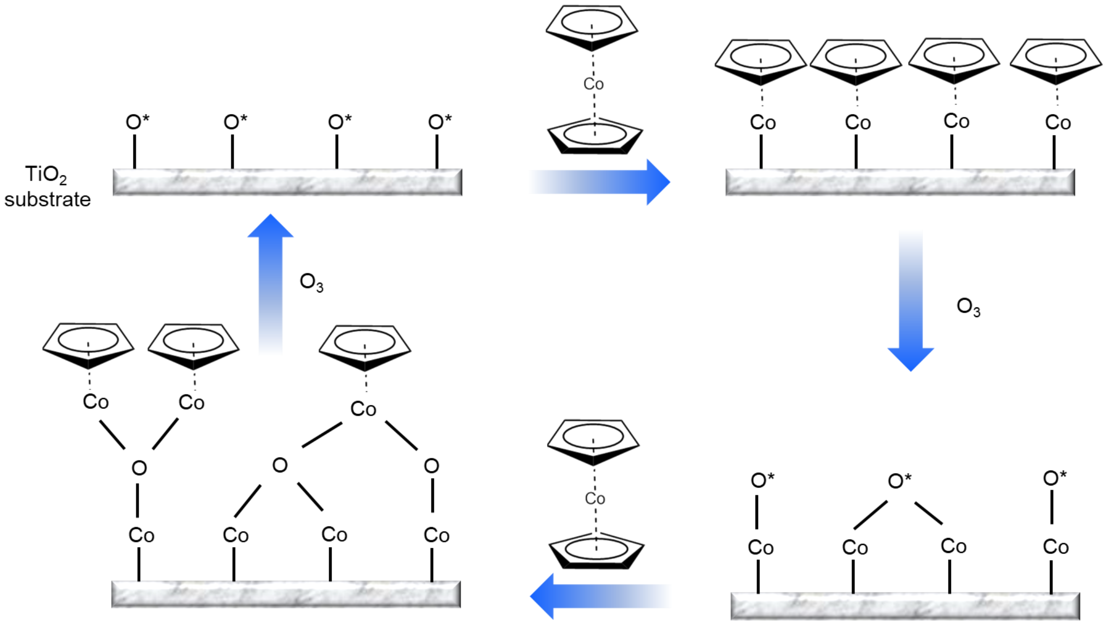

Figure S1. Typical CoO_x ALD process by using $CoCp_2$ and O_3.

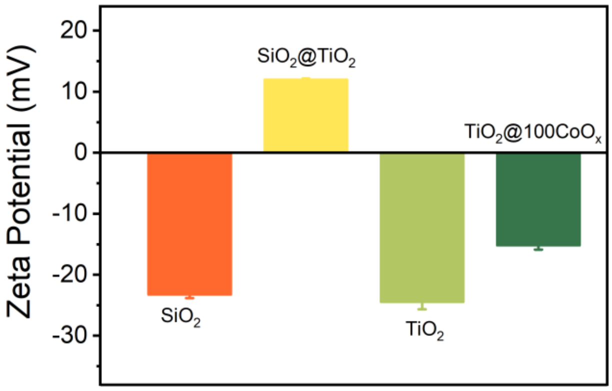

Figure S2. ζ potentials of SiO_2, $SiO_2@TiO_2$, TiO_2 and $TiO_2@100CoO_x$.

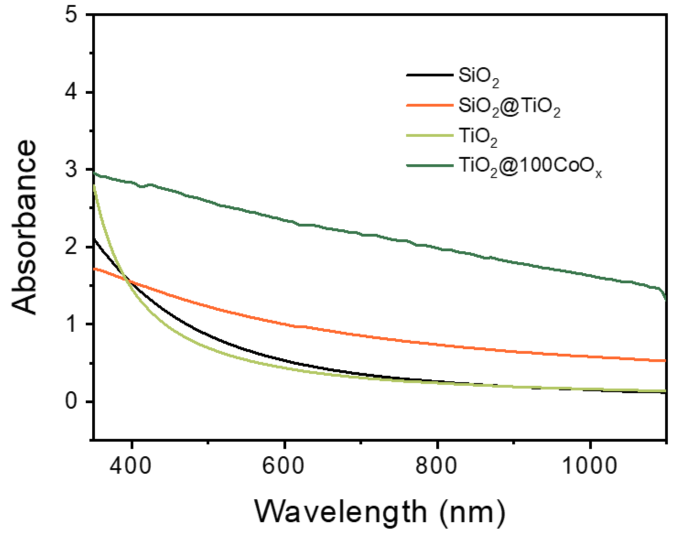

Figure S3. UV-vis spectra of SiO_2, SiO_2@TiO_2, TiO_2 and TiO_2@100CoO_x.

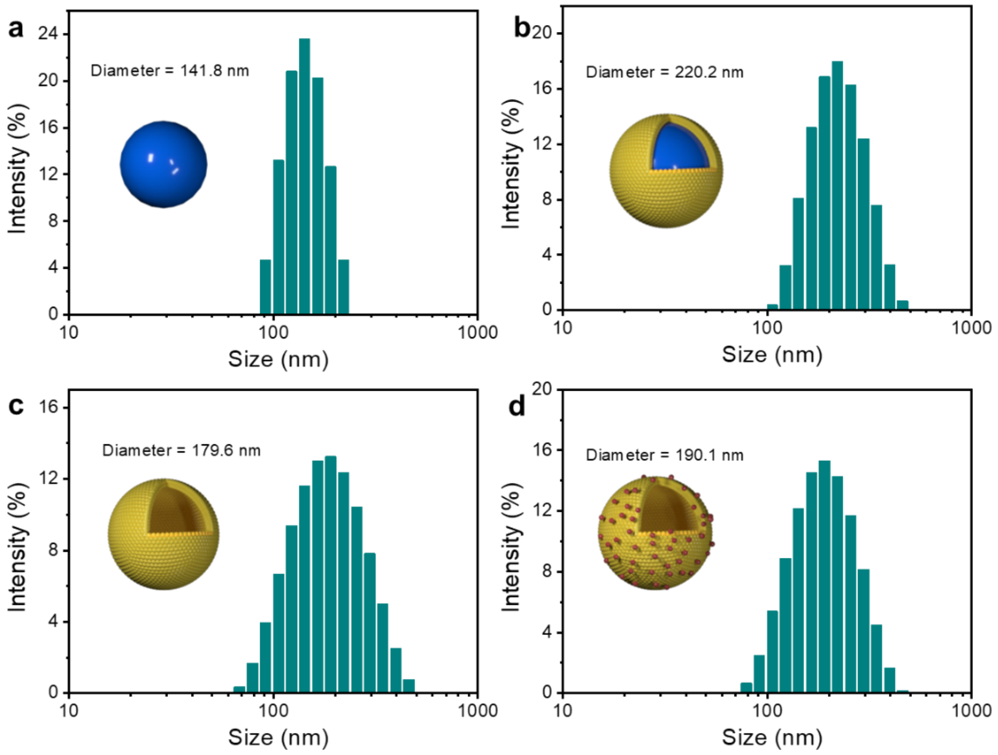

Figure S4. Particle size of (a) SiO_2, (b) SiO_2@TiO_2, (c) TiO_2 and (d) TiO_2@100CoO_x.

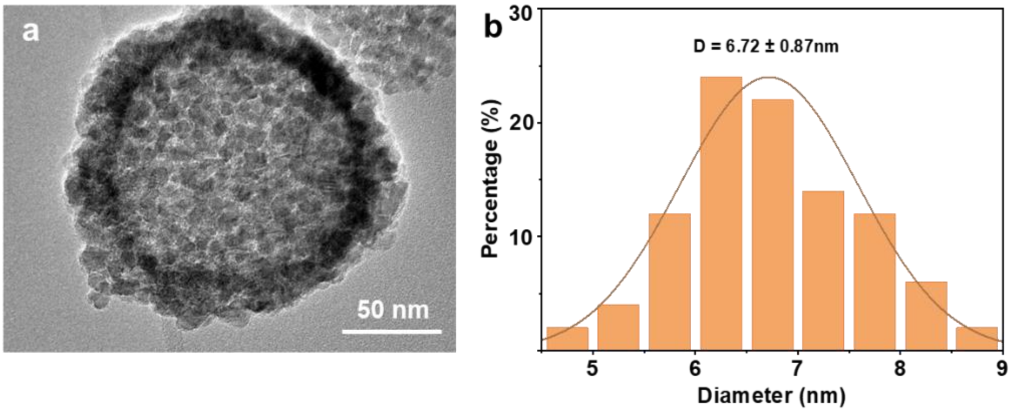

Figure S5. (a) TEM image of a hollow porous TiO_2 nanosphere. (b) Size distribution of TiO_2 grains.

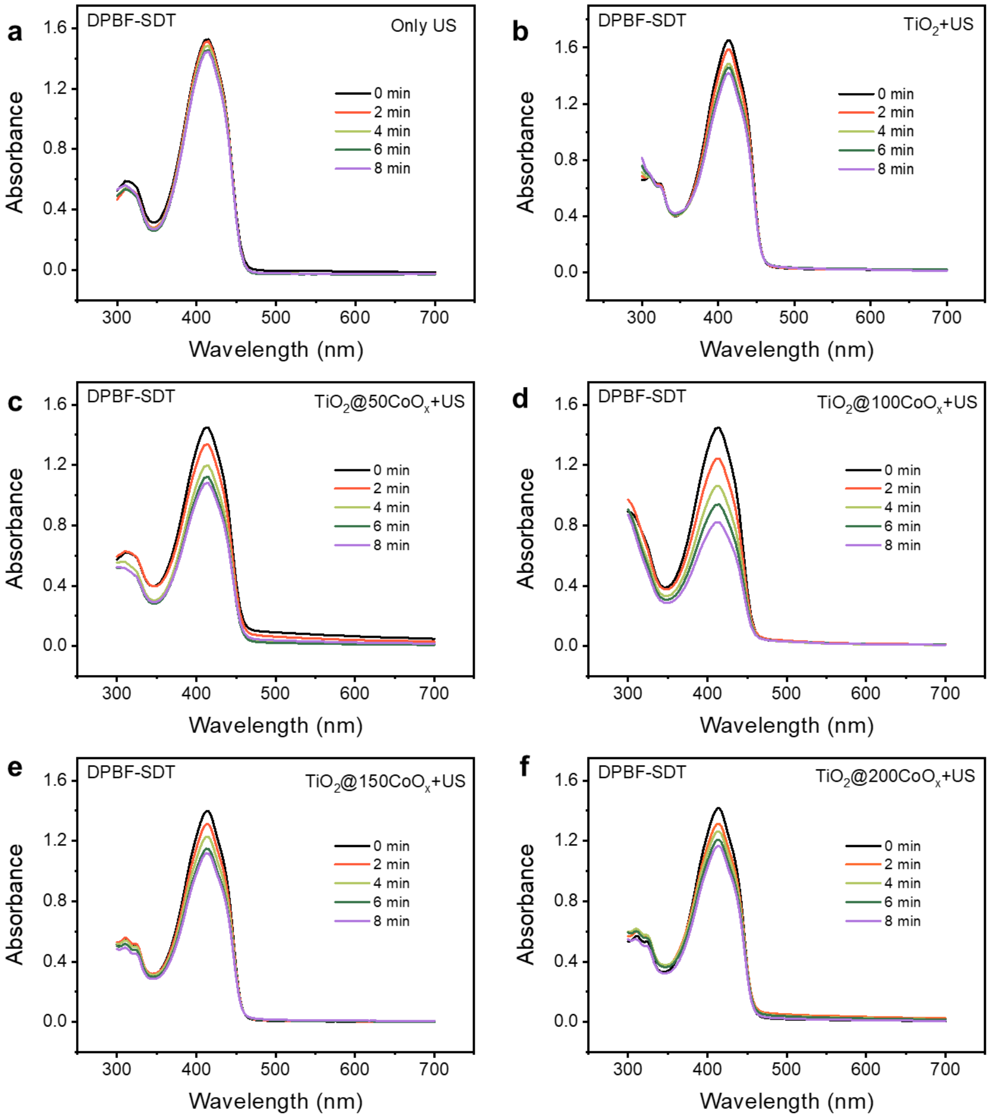

Figure S6. DPBF absorption curves treated by (a) only US, (b) TiO_2, (c) $TiO_2@50CoO_x$, (d) $TiO_2@100CoO_x$, (e) $TiO_2@150CoO_x$ and (f) $TiO_2@200CoO_x$ for the detection of $^1O_2$ in the SDT process.

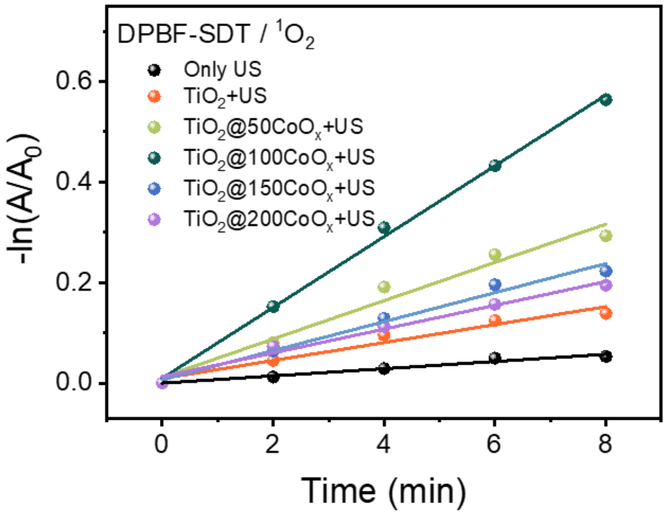

Figure S7. DPBF absorption kinetic curves by US irradiation for different durations.

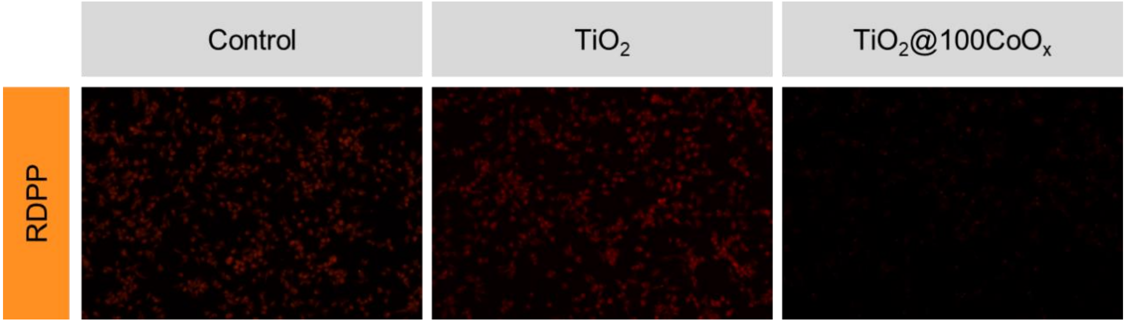

Figure S8. The images of HeLa cancer cells of different treatments after being stained with RDPP.

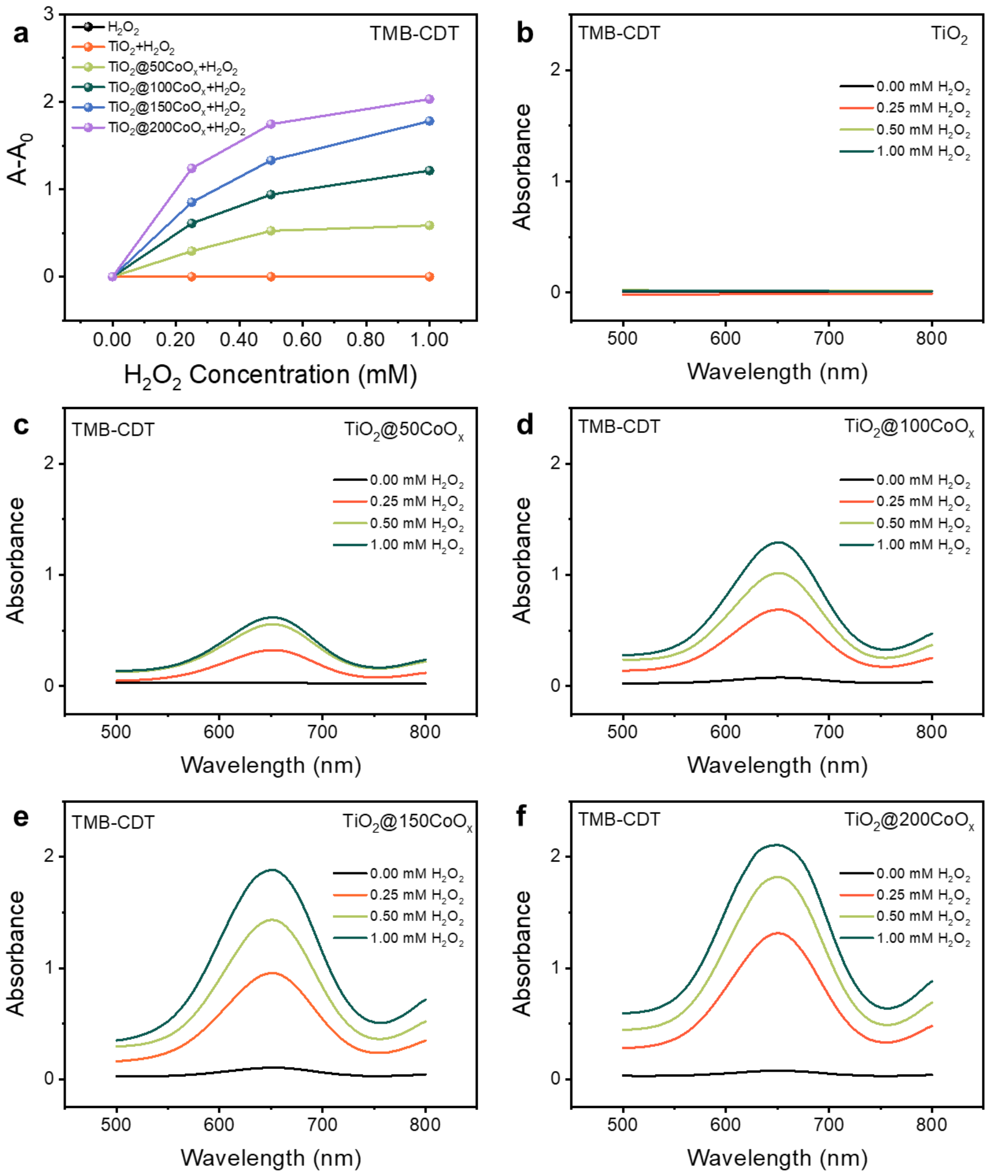

Figure S9. Generation of ROS in the CDT process measured using TMB as the probe at different H_2O_2 concentrations. (a) TMB absorbance intensity of different nanoparticles. TMB absorbance curves treated by (b) TiO_2, (c) TiO_2@50CoO_x, (d) TiO_2@100CoO_x, (e) TiO_2@150CoO_x and (f) TiO_2@200CoO_x.

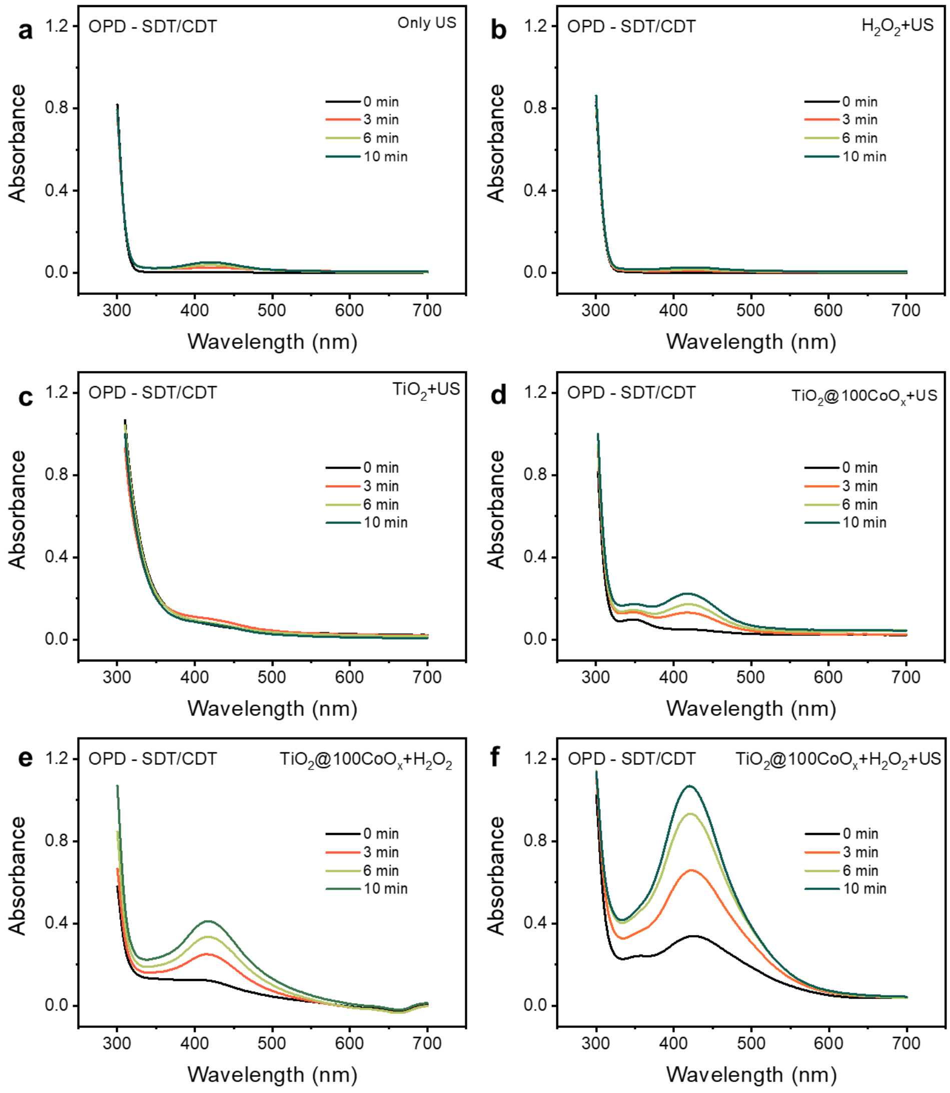

Figure S10. Production of ROS in the SDT and CDT process using OPD as the probe. Time-dependent ROS generation for (a) only US, (b) H_2O_2 + US, (c) TiO_2 + US, (d) TiO_2@100CoO_x + US, (e) TiO_2@100CoO_x + H_2O_2 and (f) TiO_2@100CoO_x + H_2O_2+ US.

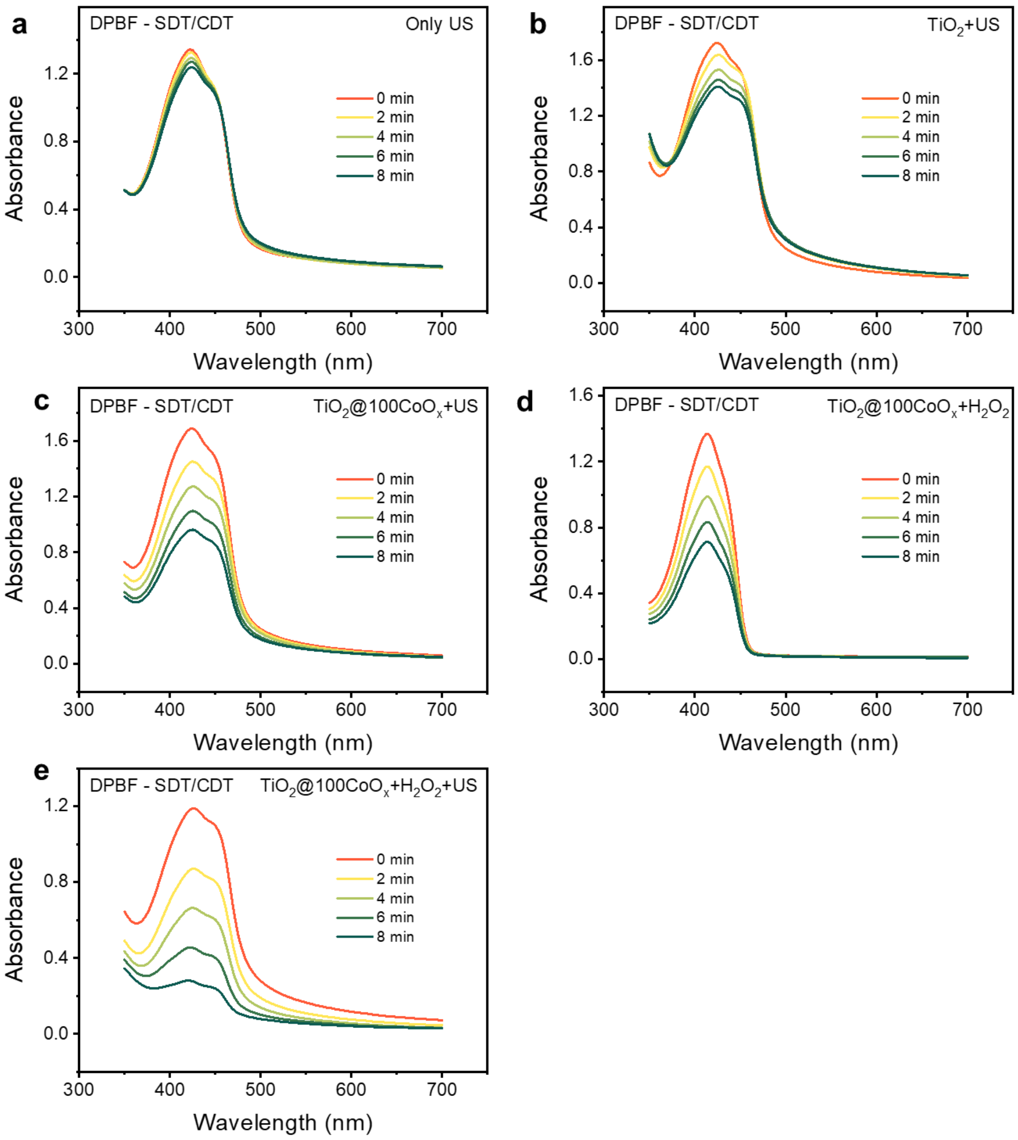

Figure S11. Production of ROS in the SDT and CDT process using DPBF as the probe. DPBF absorption curves treated by (a) only US, (b) TiO_2 + US, (c) TiO_2@100CoO_x+ US, (d) TiO_2@100CoO_x + H_2O_2 and (e) TiO_2@100CoO_x + H_2O_2 + US.

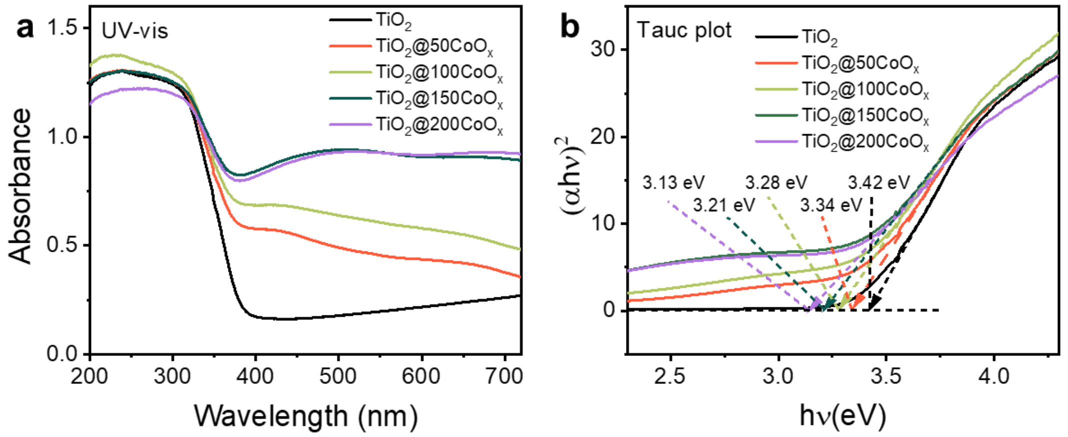

Figure S12. (a) Diffuse Reflectance Spectroscopy (DRS) and the corresponding (b) Tauc plots of the TiO_2 and TiO_2@$x$$CoO_x$.

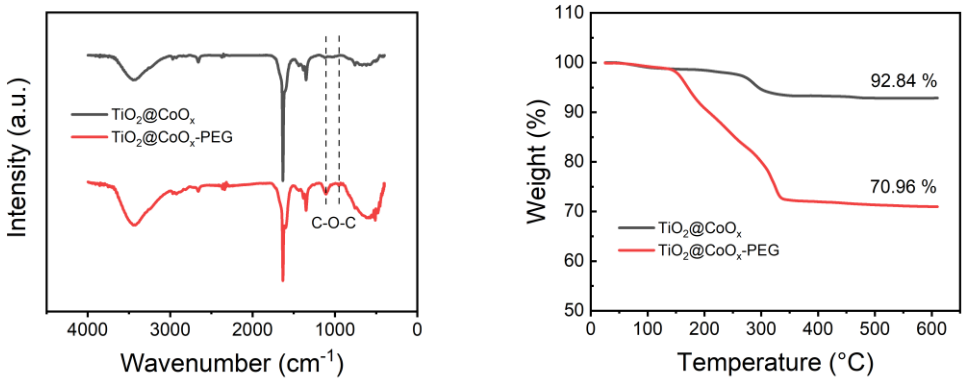

Figure S13. (a) Fourier transform infrared (FT-IR) spectra and thermogravimetric analysis (TGA) of TiO_2@100CoO_x and TiO_2@100CoO_x-PEG.

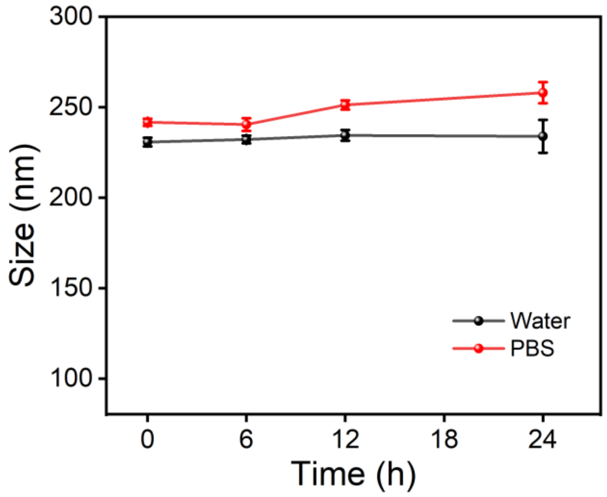

Figure S14. Time-dependent particle sizes of colloidal TiO_2@100CoO_x-PEG in water and PBS.

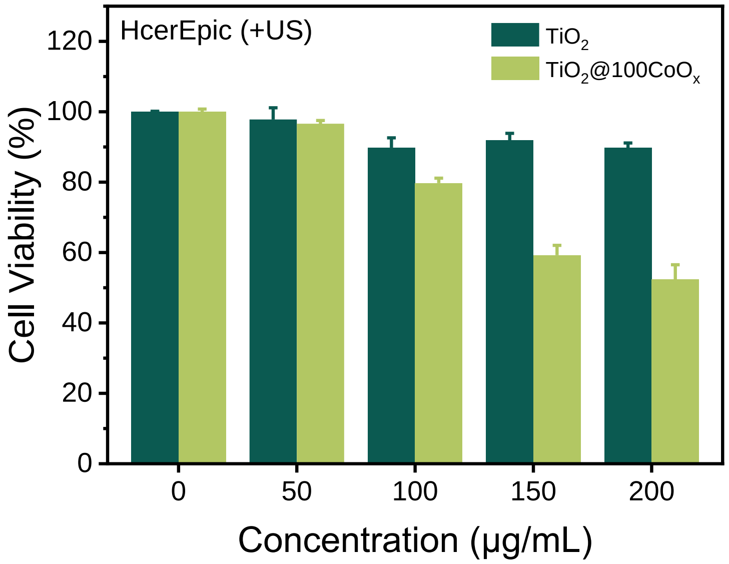

Figure S15. Relative cell viability of HcerEpic cells after incubating with TiO_2 and TiO_2@100CoO_x and US irradiation at different nanoparticle concentrations.

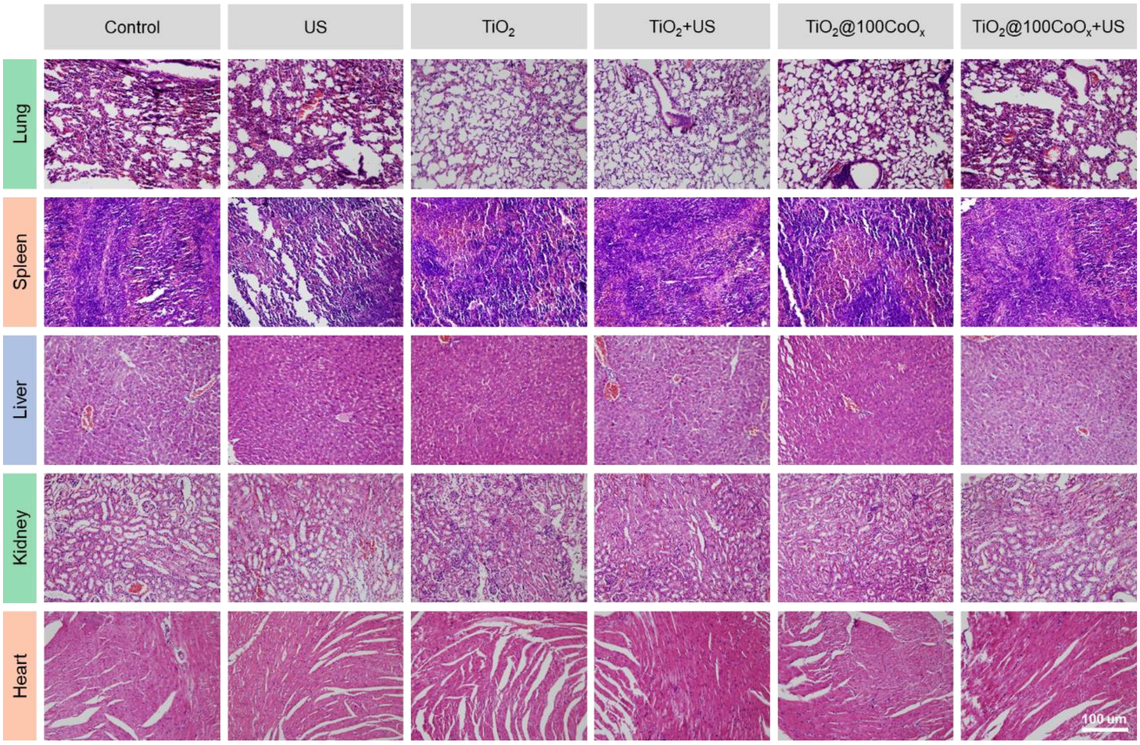

Figure S16. H&E-stained tissue sections of major organs (heart, liver, spleen, lung and kidney) dissected from mice treated by groups.

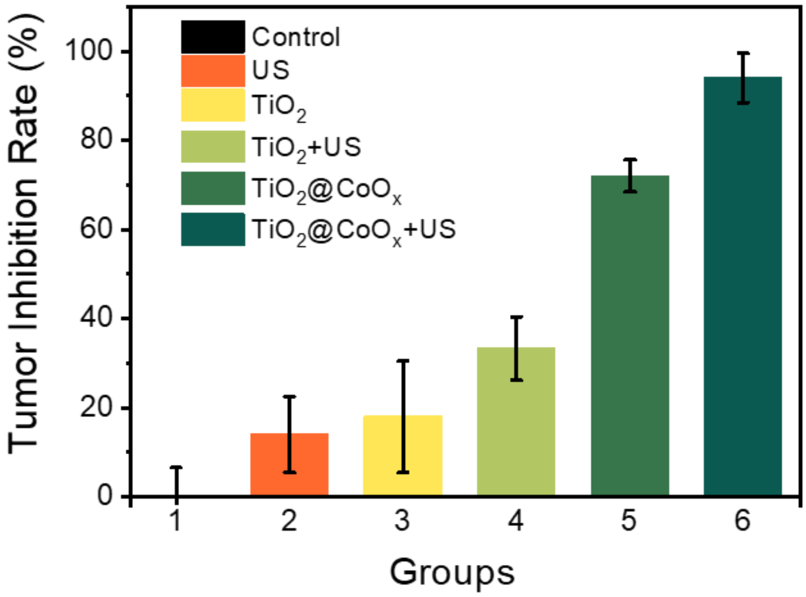

Figure S17. Tumor-inhibition rate on day 14.

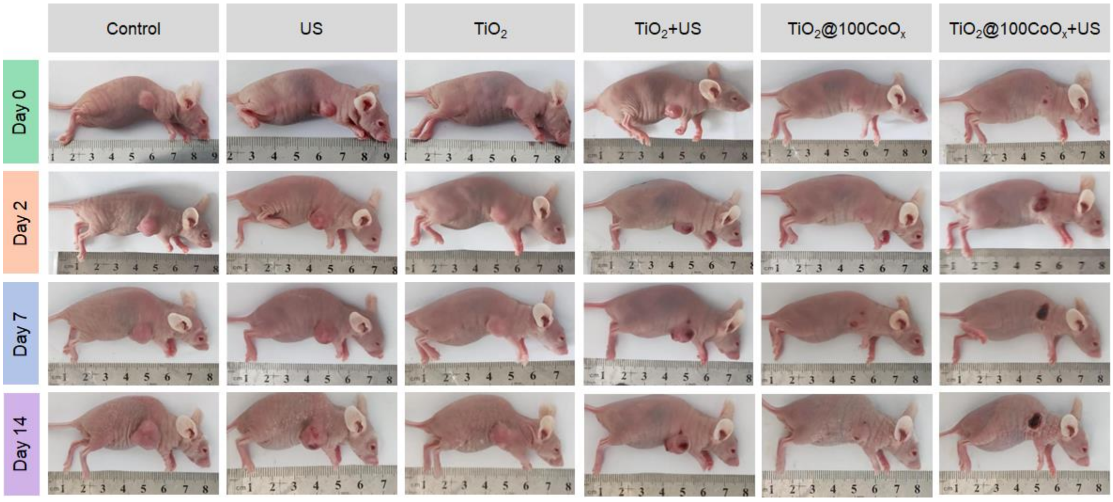

Figure S18. Digital pictures of HeLa tumor-bearing mice after different treatments for different durations (0, 2, 7 and 14 d).

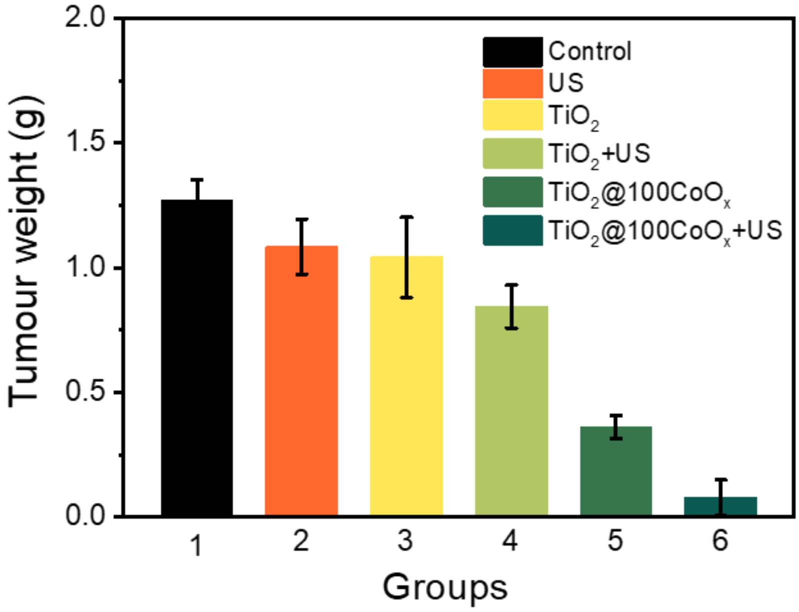

Figure S19. Tumour weight of the excised tumor on day 14.

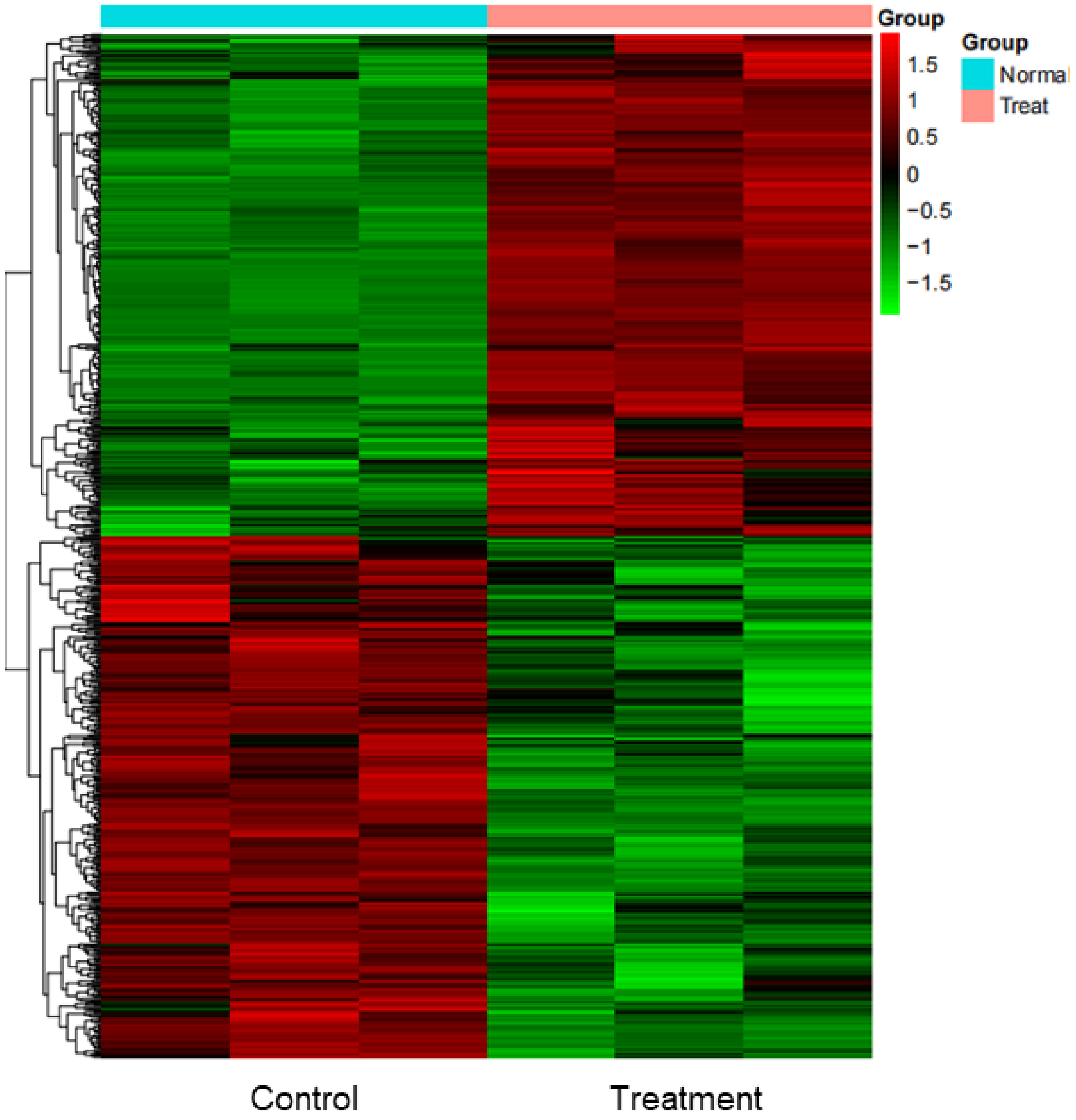

Figure S20. Clustering analysis of DEGs in control and SDT/CDT treatment of HeLa cells.

Table S1. Weight percentage from X-ray fluorescence spectrometer.

	TiO_2(wt%)	CoO_x(wt%)
TiO_2	99.99	0.01
TiO_2@50CoO_x	93.85	6.35
TiO_2@100CoO_x	88.58	12.03
TiO_2@150CoO_x	82.75	17.25
TiO_2@200CoO_x	78.82	21.18

Table S2. DPBF oxidation equation.

Nanoparticles	Oxidation equation
US	y=0.0072x+0.000
TiO_2+US	y=0.0179x+0.009
TiO_2@50CoO_x+US	y=0.0381x+0.012
TiO_2@100CoO_x+US	y=0.0704x+0.010
TiO_2@150CoO_x+US	y=0.0289x+0.007
TiO_2@200CoO_x+US	y=0.0237x+0.013
